# Exploring weighted network backbone extraction: A comparative analysis of structural techniques

**DOI:** 10.1371/journal.pone.0322298

**Published:** 2025-05-20

**Authors:** Ali Yassin, Hocine Cherifi, Hamida Seba, Olivier Togni

**Affiliations:** 1 LIB, Université Bourgogne Europe, Franche-Comté, Dijon, France; 2 ICB UMR 6303 CNRS – Université Bourgogne Europe – Franche-Comté, Dijon, France; 3 Univ Lyon, UCBL, CNRS, INSA Lyon, LIRIS, UMR5205, Villeurbanne, France; Central Asian University, UZBEKISTAN

## Abstract

Backbone extraction simplifies complex networks while retaining essential features. It reduces complexity without losing critical structural information. However, selecting the most suitable method remains challenging due to the diverse behaviors of existing techniques. This study evaluates eight structural backbone extraction methods designed for weighted networks. These methods leverage network topology rather than statistical weight distributions. A dataset of 33 real-world networks is analyzed, covering diverse sizes, topologies, and domains. Key metrics, such as Jaccard similarity and Overlap Coefficient, reveal distinct method behaviors. A hierarchical relationship emerges among methods. Primary Linkage Analysis (PLAM) captures the most substantial edges, forming the simplest backbone. Minimum Spanning Tree (MSP), Ultrametric Backbone (UMB), and Metric Backbone (MB) build on this structure, progressively adding connectivity and detail. The Doubly Stochastic Filter excels at preserving weight and degree distributions, connectivity, and transitivity. By contrast, the H-Backbone prioritizes high-weight edges but disrupts connectivity. Metric Backbone and Planar Maximally Filtered Graph ensure complete node preservation and maintain high reachability. These insights advance the understanding of structural backbone extraction techniques for weighted networks. They benefit applications in fields like biology, social networks, and transportation. Practitioners can better achieve goals like network simplification for visualization or property preservation for analysis.

## 1 Introduction

In recent years, the study of complex systems has witnessed a paradigm shift with the emergence of network analysis as a powerful tool [1–4]. Networks, comprised of interconnected nodes and edges, provide a comprehensive framework for understanding the intricate interactions within these systems [5–18]. Incorporating numerical weights on edges has given rise to weighted networks, offering a nuanced perspective on the intensity of node interactions [19].

The computational efficiency of many network algorithms relies on the sparsity principle, assuming that networks exhibit a certain degree of sparsity. While setting a threshold on edge weights is a common approach to reducing network size, it often sacrifices the natural diversity of edge weight distribution. Researchers have developed techniques to extract network backbones to address this challenge, preserving essential characteristics while downsizing the overall structure.

Backbone extraction has proven to be a valuable tool across a wide range of domains and applications. For instance, in biological networks, such as protein interaction and metabolic networks, backbones help identify key functional interactions. They also reveal regulatory mechanisms critical for understanding cellular behavior and disease pathways [8, 20, 21]. In transportation networks, backbone techniques optimize route planning, connectivity analysis, providing insights into critical infrastructure and traffic flow [22–25].

Similarly, in finance, backbone extraction methods are used to analyze systemic risk by identifying the most significant channels of contagion between financial institutions [26–28]. In social networks, backbones uncover the core structure of relationships, enhancing the understanding of information diffusion, opinion dynamics, and community structures [29–32]. Additionally, in ecological networks, backbones highlight the energy flow within food webs and help identify keystone species critical for ecosystem stability [33, 34].

Recent advances demonstrate the adaptability of backbone extraction techniques to novel applications. For example, in misinformation studies, network backbones reveal the structural dynamics of misinformation spread across digital platforms, such as WhatsApp and Facebook, aiding in combating fake news [31, 35]. In global environmental networks, backbone approaches help track international collaborations and highlight central actors critical for environmental governance [36]. Furthermore, in neuroscience, backbone techniques elucidate brain connectivity and its relationship with cognitive performance and mental health [37, 38].

These techniques fall into two main categories: statistical and structural methods. Statistical techniques use hypothesis testing or empirical weight distribution to evaluate the significance of each edge. The Disparity filter, Polya Urn filter, Noise Corrected Filter, Marginal Likelihood filter, ECM filter, GloSS filter, and LANS filter represent diverse approaches within this category.

Hypothesis testing involves generating p-values by contrasting observed edge weights with a null model. The p-values quantify the deviation from the null hypothesis. In contrast, empirical weight distribution-based techniques assess edge significance by comparing observed weights against the overall weight distribution.

On the other hand, structural techniques extract a backbone with specific topological properties by operating on the network’s topology. These methods can be broadly categorized into two categories: methods that extract a single substructure and methods that assign scores to nodes or edges. The latter category offers greater flexibility by adjusting threshold levels.

Researchers have extensively compared statistical methods in previous studies, providing valuable insights into their strengths, limitations, and applicability across various network contexts . Furthermore, several studies have explored the comparative performance of statistical and structural methods, highlighting the distinctive characteristics of these approaches and their suitability for specific types of networks. Researchers have extensively compared statistical methods in previous studies. They provide valuable insights into their strengths, limitations, and applicability across various network contexts [39–41]. Specifically, A recent study [42] systematically compares seven statistical hypothesis-testing backbone extraction methods across diverse networks, including Disparity Filter (DF) [43], Polya Urn Filter (PF) [44], Marginal Likelihood Filter (MLF) [45], Noise Corrected (NC) [46], Enhanced Configuration Model Filter (ECM) [47], Global Statistical Significance Filter (GloSS) [48], and Locally Adaptive Network Sparsification Filter (LANS) [49]. The study evaluates extracted backbones based on similarity, edge-feature correlations, global properties, and edge and degree distributions. It highlights the unique characteristics of these methods, such as DF and LANS favoring high-weight edges, ECM assigning lower significance to high-degree edges, and the PU filter’s strong preservation of weight distribution. Notably, the study reveals a hierarchical relationship among several methods, showing that backbones extracted using methods like NC and MLF encapsulate those of preceding techniques. This work underscores the importance of choosing an appropriate backbone method to preserve specific network properties and provides practical guidance for applications in weighted network analysis.

On the other hand, several studies have explored the comparative performance of statistical and structural methods. They highlight the distinctive characteristics of these approaches and their suitability for specific types of networks.

A prior investigation [50] examines six backbone extraction techniques in the Southeast Asian intercity air transport network. It covers five structural approaches (global weight thresholding, k-core decomposition [51], minimum spanning tree [52], primary linkage analysis [53], and multiple linkage analysis [54]) and one statistical method (the disparity filter) [43]. The study evaluates the extracted backbones based on geographical and topological structures. It highlights the utility of each technique for different transport research applications. K-core decomposition identifies well-connected cores in multiplex networks. Primary linkage analysis reveals hub-and-spoke configurations and functional regions. The disparity filter uncovers hidden structures and offers a comprehensive view of large-scale networks’ topological and spatial information.

Gomes Ferreira *et al*. [55] propose a four-step principled methodology for comparing and selecting the most appropriate backbone extraction method. This methodology is tailored to a given phenomenon of interest. This analysis includes using two structural methods (Global Thresholding, and High Salience Skeleton [56]) and eight statistical methods (RECAST [57], Disparity [43], Polya Urn [44], Marginal Likelihood [45], Noise Corrected [46], Global Statistical Significance, [48], Tripartite Backbone Extraction [58], and Stochastic Degree Sequence Model filters [59]). They validate their approach in two case studies: online discussions on Instagram and coordinated behavior in WhatsApp groups. Results show that each method can produce very different backbones. This underlines that choosing an adequate method is of utmost importance to reveal valuable knowledge about the particular phenomenon under investigation.

We evaluate six backbone extraction techniques in the context of the American Elementary School contact network [60] . This analysis included two statistical methods (Disparity Filter [43], Locally Adaptive Network Sparsification (LANS) [49]), three structural methods (Doubly Stochastic [61], High Salience Skeleton [56], Metric Backbone [62]), and one hybrid method (Globally and Locally Adaptive Network Backbone (GLANB) [63]). Thre results reveale distinct influences of each technique on the network’s community structure. For example, Disparity and LANS methods results in multi-component backbones. While the Doubly Stochastic method preserves transitivity and maintains a balanced ratio of intra- and inter-community edges. In contrast, other techniques emphasizes intra-community links. The GLANB method stood out for its filtering precision and accurate representation of the network’s community structure.

Despite these contributions, to the best of our knowledge, no prior work has focused exclusively on a comprehensive comparison of structural backbone extraction methods. Our preliminary work [64] addressed this gap by evaluating eight structural backbone extraction techniques in the context of the World Air Transportation network. First, the study examines the similarity between the extracted backbones. This is done by computing the Jaccard similarity scores for pairs of extracted backbones. Results reveal that shortest-path-based methods produced similar backbones, whereas the Doubly Stochastic and H-Backbone methods exhibit distinct and uncorrelated behaviors.

Then we examine the relationship between backbone edge inclusion and edge weight. Our findings show that the H-Backbone method predominantly retains high-weight edges. While the High Salience Skeleton and Doubly Stochastic methods capture a broader range of weight scales.

To quantify information loss resulting from the filtering process, we compare basic topological properties of the extracted backbones including: edge, node, and weight fractions. The Doubly Stochastic and H-Backbone methods preserve a significantly higher proportion of edges compared to other techniques. However, methods like the H-Backbone, High Salience Skeleton, and Doubly Stochastic fail to retain all nodes in the network.

Finally, we evaluate the backbone connectivity and transitivity by analyzing metrics such as reachability, the number of components, the size of the largest connected component, and transitivity. Results highlight that the Primary Linkage Analysis and High Salience Skeleton methods disrupt network connectivity. While the Doubly Stochastic method demonstrates superior performance in preserving transitivity.

However, the scope of this work was limited to a single network and a narrow range of properties. By building on our preliminary work, this study makes the following key contributions:

- **Analysis Across a Diverse Dataset:** This study extends previous work by employing a significantly larger and more diverse dataset. The dataset encompasses networks from various domains and of varying sizes. The expansion enhances the generalization of the findings. Consequently, they become applicable to a wider range of real-world scenarios, including social, biological, and infrastructural networks.
- **Similarity Analysis:** We begin by analyzing the similarities between different filtering techniques using the Jaccard Similarity score [65] and the Overlap Coefficient [66]. This experiment provides researchers with insights into which techniques may yield comparable results and which are more distinct.
- **Backbone Edge Characteristics:** Using the Point Biserial correlation [67] we examine the relationships between backbone edge inclusion and local edge properties such as: weight, degree, weighted degree, betweenness, and weighted betweenness. This analysis helps in understanding the sensitivity of backbone extraction methods to network local properties.
- **Comparison of Global Backbone Properties:** Adopting a macro-level view, we compare global backbone properties across the filtering techniques. This includes evaluating edge proportions, nodes, weights, weight entropy, reachability, number of components, and transitivity. These metrics provide a comprehensive view of how each technique impacts the network structure, offering valuable insights into their effectiveness in preserving critical properties.
- **Comparison of Backbone Distributions:** Finally, we compare the weight and degree distributions of the extracted backbones against the original networks using the two-sample Kolmogorov-Smirnov test (KS test) [68]. This experiment highlights how well each method captures the original network’s diversity in connectivity and interaction strength.

Each of these contributions advances the field by addressing critical gaps, providing a comprehensive understanding of structural backbone filtering techniques, and equipping researchers and practitioners with the knowledge to make informed choices tailored to their objectives.

The paper is organized as follows: Sect 2 introduces the structural backbone extraction methods. Sect 3 provides an overview of the dataset used in the study. Sect 4 examines the similarities between techniques using the Jaccard Similarity score and Overlap Coefficient. Sect 5 investigates the relationship between backbone edges and local edge properties, such as weight, degree, and betweenness. Sect 6 evaluates the global topological properties of the extracted backbones. Sect 7 analyzes the weight and degree distributions of the backbones. The results are discussed in Sect 8, followed by the conclusions in Sect 9. Finally, the Supporting information details the data, materials and evaluation measures of this study.

## 2 Structural backbone extraction methods

This section introduces the structural backbone extraction techniques evaluated in this study. These methods were selected based on several criteria. First, we prioritize weighted methods, as there has been limited prior work comparing weighted methods in weighted networks. Second, we focus on edge-based methods, as they directly target the relationships between nodes. Lastly, we include techniques frequently used in previous surveys and those recognized as the most popular in the field. The selected methods can be categorized into two groups: single-structure extraction methods and score-based extraction methods.

For more detailed information about each method, including their descriptions, advantages, disadvantages, and computational complexities, please refer to Table 1. This summary provides a concise comparison of the methods and highlights the trade-offs associated with each technique.

**Table 1:** Summary of the reviewed backbone extraction methods.

Method	Description	Scope	Advantages	Disadvantages	Complexity
Maximum Spanning Tree (MSP) [52]	Extracts a subgraph connecting all nodes without cycles, maximizing total edge weight.	Global	Includes all nodes; emphasizes high-weight edges.	May oversimplify complex networks.	
Planar Maximally Filtered Graph (PMFG) [69]	Iteratively adds the highest-weight edges while maintaining graph planarity.	Global	Maintains planarity; emphasizes high-weight edges.	Limited to planar networks; may exclude meaningful connections in dense networks.	O(ElogE+E): Sorting edges and ensuring planarity constraints.
Primary Linkage Analysis (PLAM) [70]	Retains the highest-weight edge for each node.	Local	Includes all nodes; Emphasizes most significant connections per node.	Ignores global network structure	O(N+E): Loops through all nodes and their edges to find the highest-weight edge per node.
h-Backbone (HB) [71]	Combines edge weights, connectivity, and betweenness centrality to construct the h-strength and h-bridge.	Global	Incorporates multiple topological properties.	May Isolate many nodes, and exclude weak but important edges.	$O(N\cdot E+N^{2}logN)$: Dominated by edge betweenness centrality computation.
Metric and Ultrametric Backbones (MB and UMB) [62]	Extract shortest-path subgraphs, with Metric using the sum of distances and Ultrametric using maximum distance.	Global	Captures shortest-path structures; provides flexibility in path definitions.	May exclude meaningful non-shortest-path edges; sensitive to edge distance definitions.	
Doubly Stochastic (DS) [61]	Converts adjacency matrix into a doubly stochastic matrix, sorting and retaining edges with highest scores.	Local	Ensures single connected component; emphasizes high-score edges.	Transformation may fail for certain networks; sensitive to normalization process.	$\text{O}({\text{V}}^{3})$: Iterative row/column normalization until convergence.
High Salience Skeleton (HSS) [56]	Retains edges with high salience score near 1, representing their presence in shortest-path trees.	Global	Avoids arbitrary thresholds; identifies edges crucial to shortest paths.	May exclude edges with moderate salience that are globally important.	$O(V\cdot E+V^{3})$: Computing shortest paths for all nodes and calculating edge salience.

### 2.1 Single-structure extraction methods

These methods extract a single substructure from the network without the ability to adjust or tune parameters.

#### Maximum Spanning Tree Filter (MSP) [52]:

This method extracts a subgraph that connects all nodes without forming cycles, maximizing the total edge weight. It ensures that all nodes are included while focusing on edges that maximize the network’s cumulative weight.

#### Planar Maximally Filtered Graph (PMFG) [69]:

This method operates by iteratively reconstructing the graph. It adds edges with the highest weight one at a time, ensuring the resulting graph remains planar throughout the process. This approach balances edge retention with structural simplicity.

#### Primary Linkage Analysis (PLAM) [70]:

For each node, this method selects the edge with the highest weight, creating a backbone that retains the most influential connections based on edge weights.

#### h-Backbone Filter (HB) [71]:

Inspired by the h-index, this method combines edge weights, connectivity, and betweenness centrality. The procedure involves the extraction of an h-strength network, where h is the largest natural number such that there are h links, each with a weight at least equal to h. Subsequently, the method extracts an h-bridge network similarly. Here, the bridge of an edge is determined by dividing its edge betweenness by the total number of nodes in the network. Finally, the h-backbone is obtained by merging the information from the h-strength and h-bridge networks.

#### Metric and Ultrametric Distance Backbone Filters (MB and UMB) [62]:

Both methods extract subgraphs consisting of shortest paths but differ in their definitions of path length. The Metric filter defines it as the sum of edge distances, while the Ultrametric filter uses the maximum edge distance in the path, resulting in different extracted subgraphs.

### 2.2 Score-based extraction methods

These methods assign scores to edges or nodes based on topological properties and extract the backbone by applying thresholds or selecting a fraction of scores, allowing for tuning and customization.

#### Doubly Stochastic Filter (DS) [61]:

This method transforms the adjacency matrix into a doubly stochastic matrix through iterative normalization of row and column sums. The normalized weights are consideres the new scores. Then edges are sorted by normalized weights in descending order and sequentially added to the backbone until all nodes form a single connected component. Note that the transformation may not always be feasible for some networks.

#### High Salience Skeleton Filter (HSS) [56]:

This method computes edge salience based on the proportion of shortest-path trees. Initially, a shortest path tree is constructed for each node by merging all the shortest paths from that node to every other node in the network. The edge salience is then computed, representing the proportion of shortest-path trees in which a given edge is present. The authors observed that edge salience follows a bimodal distribution near the boundaries 0 and 1. Consequently, they retain only the edges with salience near 1.

## 3 Data and methods

This section outlines the data and methods for each experiment.

### 3.1 Data

The data used in the experiments consists of 33 real-world networks spanning diverse domains, including character, web, social, biological, infrastructural, and economic networks. This dataset was chosen to ensure a comprehensive evaluation of the backbone extraction techniques across a variety of network types, sizes, and topologies. The diversity in density, average clustering coefficient, and node count provides a robust foundation for assessing the generalizability and adaptability of the methods. In addition, many of these networks have been used in previous surveys [42], adding credibility and comparability to the results.

**Fig 1:**
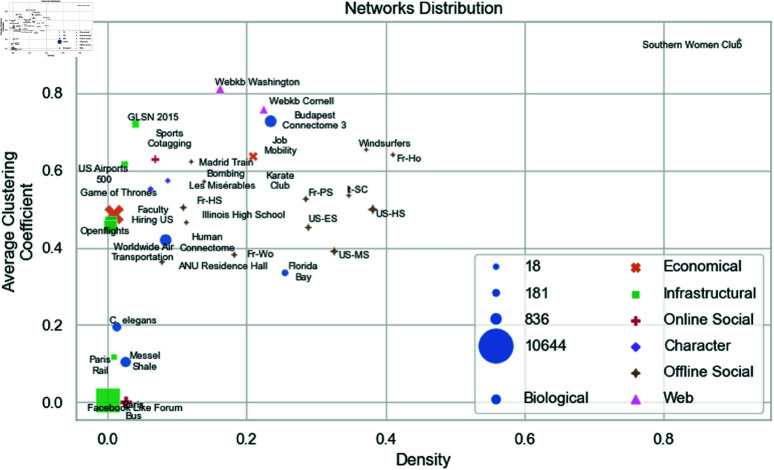
A scatter plot illustrating the distribution of networks based on density and average clustering coefficient. Marker sizes represent the number of nodes in each network.

Fig 1 illustrates the dataset’s diversity using a scatter plot, where density and average clustering coefficient are represented on the axes, and marker sizes indicate the number of nodes in each network. This visualization highlights the variation in structural characteristics across the networks. We refer to the Supporting information for a detailed description of each network.

#### Character networks:

Includes networks such as the Les Misérables [72] and the Game of Thrones network [73] which represents the occurrences of the characters of these books.

#### Biological networks:

Includes networks such as the Budapest Connectome [74, 75], Human Connectome [76], C. elegans [77], Messel Shale [78], and Florida Bay [79] networks, each representing different biological systems such as brain connectivity, genetic interactions, and ecological food webs.

#### Economic networks:

Consists of the Faculty Hiring US [80] and Job Mobility (https://www.michelecoscia.com/?page_id=312) networks, which depict patterns of academic hiring and job transitions, respectively.

#### Infrastructural networks:

Encompass networks like Worldwide Air Transportation [81, 82], Openflights [83], US Airports 500 [84], Paris Bus [85], Paris Rail [85], and GLSN 2015 [86], representing various infrastructural systems such as air transportation, public transportation, and international shipping routes.

#### Offline social networks:

Comprise networks such as the Davis’ Southern Women Club[87], Karate Club [88], Madrid Train Bombing Terrorists [89], Windsurfers [90], Illinois High School Students [91], ANU Residence Hall [92], American High School [93], American Middle School [94], American Elementary School [94], French Primary School [95], French High School [95], Workplace [95], ACM Hypertext 2009 Scientific Conference [95], and Geriatric Ward of French Hospital [95] networks, depicting social interactions in different offline contexts including schools, workplaces, and events.

#### Online social networks:

Include networks like Facebook Like Forum [96] and Sports Cotagging [97] representing interactions among users or topics in online platforms.

### 3.2 Methods

This subsection outlines the methods and procedures employed in our experiments.Note that the experiments are conducted on a machine equipped with an Intel Core i9-11950H. We used Python 3.10 and the Netbone package [98] (version 0.2.3) to extract and evaluate the backbones.

#### 3.2.1 Experiment 1: Exploring technique similarities.

The primary objective of the initial experiment is to unveil similarities among various backbone filtering techniques, employing two metrics: the Jaccard similarity (*J*) and the Overlap Coefficient (*Ov*).

The first step involves extracting the backbones in each network *n* using different backbone extraction methods. For each pair of backbones (*B*_1_ and *B*_2_), the set of edges (*E*_1_ and *E*_2_) is obtained, and the Jaccard similarity coefficient ($J(n,E_{1},E_{2})$) is computed. Subsequently, the results are aggregated by calculating the mean ($\mu_{\left(E_{1},E_{2}\right)}$) and standard deviation ($\sigma_{\left(E_{1},E_{2}\right)}$) across all networks using the following formulas:

$$\begin{array}{ll} \mu_{\left(B_{1},B_{2}\right)}= & \frac{1}{N}\sum_{n=1}^{N}J(n,B_{1},B_{2})\\ \sigma_{\left(B_{1},B_{2}\right)}= & \sqrt{\frac{\sum_{n=1}^{N}{\left(J(n,B_{1},B_{2})-\mu_{\left(B_{1},B_{2}\right)}\right)}^{2}}{N}} \end{array}$$ (1)

In the second step, the Overlap Coefficient is computed based on the perspective of each set ($Ov_{B1}(n,E_{1},E_{2})$ and $Ov_{B2}(n,E_{1},E_{2})$). Similar to the first procedure, the results are aggregated, generating a heatmap for visual representation.

#### 3.2.2 Experiment 2: Exploring the relation between the backbone edges and the edge properties.

This experiment explores the relationship between backbone edges and edge properties, such as weight, degree, and betweenness, and their weighted versions. Since not all the methods assign scores to the edges, an extension of the Pearson correlation coefficient [99] is used. The Point Biserial Correlation coefficient is often used when one of the variables is dichotomous (having only two categories, often coded as 0 and 1), which in our case is the presence and absence of an edge in the backbone.

The analysis aims to uncover how the statistical significance of edges relates to specific network attributes, providing insights into the behavior and nature of the selected backbone edges. By investigating these associations, we can determine whether certain edge properties are more likely to be retained or discarded during the filtering process, thus offering a nuanced understanding of the decision-making mechanisms employed by filtering techniques.

The procedure starts by extracting the backbones for each network *n* using the different backbone extraction methods. Then, we assign the presence scores *P*_*B*_ for each edge: a value 1 to an edge if it is present in the backbone and 0 otherwise.

Subsequently, for each property *Y*, we compute the edge property *Y* for every edge in the original network. Following this, Point Biserial correlation coefficients $r_{P_{B},Y}$ are determined between the edge presence scores *P*_*B*_ for each backbone *B* and each edge property *Y*.

Finally a violin is generated for each edge property *Y* to visualize the Point Biserial correlation coefficients distribution for each method across all networks.

#### 3.2.3 Experiment 3: Exploring the backbones global properties.

This experiment comprehensively examines global characteristics in extracted backbones through various techniques. The objective is to discern these methods’ impact on networks’ topological attributes. Additionally, we endeavor to evaluate how the network’s size influences the efficacy of backbone extraction methods in terms of global topological properties.

The experimental procedure commences with extracting backbones for each network *n* using diverse backbone extraction methods. Subsequently, we compute the value of each topological property (*Y*), denoted as *P*(*Y*,*B*), for each backbone *B*. To contextualize these values, we normalize them by the corresponding values of the original network (*O*) using the equation:

$$PN_{Y,B}=\frac{P_{\left(Y,B\right)}}{P_{\left(Y,O\right)}}$$ (2)

To facilitate the visualization of results, we depict the normalized values in a violin plot for each backbone extraction method.

#### 3.2.4 Experiment 4: Comparing backbones distributions.

In the previous experiment, we explore the overarching characteristics of backbones and revealed the attributes associated with each method of backbone extraction. In this iteration, we replicate the same methodology focusing on distributions, specifically those about weight and degree. The experimental process is initiated by extracting backbones for each network *n* using diverse extraction methods. Subsequently, we extract the weight and degree values of both the extracted backbones (*D*_*w*_(*B*) and *D*_*d*_(*B*)) and the original network (*D*_*w*_(*O*) and *D*_*d*_(*O*)). Due to differences in backbone sizes, the distribution sizes also vary. Consequently, we opt to employ the Kolmogorov-Smirnov test for comparison. The Kolmogorov-Smirnov statistic ($KS(D_{w}(B),D_{w}(O))$) and ($KS(D_{d}(B),D_{d}(O))$) are computed for each backbone distribution in comparison to the original network.

For each property (weight, degree), the distribution of KS values is visually presented in a violin plot for each method of backbone extraction.

Subsequently, the methods are ranked based on the KS statistic values in each network, assigning a rank of 1 to the method closest to the original network and 8 to the method farthest away. Finally, the results are depicted in a box plot. It’s important to note that the experiment used networks with a minimum of 1000 edges.

## 4 Experiment 1: Exploring technique similarities

This section reveals commonalities among backbone filtering techniques using two complementary measures: the Jaccard score and the Overlap Coefficient. The procedure initiates in each network by extracting the backbones using various backbone extraction methods. Subsequently, the edge sets of each pair of methods are used to calculate the Jaccard score and Overlap Coefficient in each network. The results are then aggregated, and the mean and standard deviation are computed across all networks for each pair. This comprehensive approach provides insights into the similarities and overlaps between backbone extraction methods across diverse networks.

### 4.1 Jaccard similarity score

**Fig 2:**
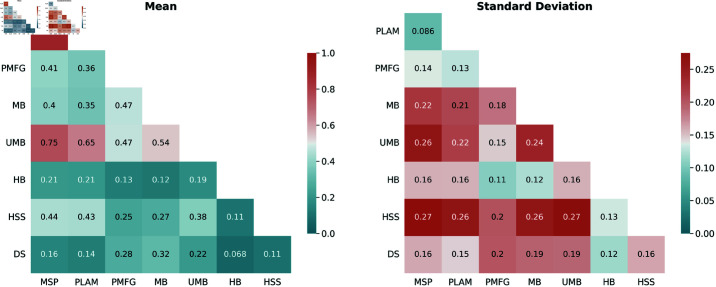
The heatmaps of the Jaccard similarity score mean, and standard deviation between edges sets for different backbone pairs.

Fig 2 displays heatmaps illustrating the mean and standard deviation of Jaccard similarity scores between sets of edges for various backbone filtering techniques. The analysis reveals significant correlations among specific pairs of techniques, such as the strong correlations between Primary Linkage Analysis and Minimum Spanning Tree Filters (PLAM-MSP) and Ultrametric Backbone and Minimum Spanning Tree Filters (UMB-MSP).

The H-Backbone and Doubly Stochastic Filters stand out for their lack of correlation with other techniques, indicating distinct behavior. The low standard deviation in the heatmaps adds credibility to the observed correlations and behaviors, highlighting the consistency and reliability of the findings. Most other techniques show network-specific behaviors without consistent patterns across all networks. The study emphasizes the diverse behavior of backbone filtering techniques, with each method responding uniquely to network characteristics.

### 4.2 Overlap coefficient

**Fig 3:**
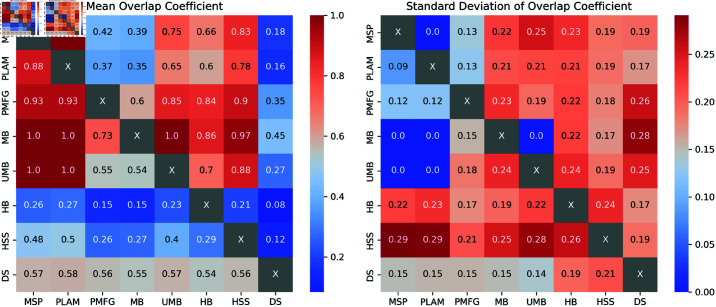
The heatmaps illustrate the mean and standard deviation of the overlap coefficient between sets of edges for various backbone pairs. As the overlap coefficient is calculated relative to each method within the pairs, the resulting heatmap is asymmetrical.

Fig 3 presents heatmaps depicting the mean and standard deviation of the Overlap Coefficient between sets of edges for various pairs of backbones. Key observations from the analysis include:

#### Total Overlap Among Methods:

The Primary Linkage Analysis Backbone is included in the Minimum Spanning Tree, the Minimum Spanning Tree Backbone is included in the Ultrametric Backbone, and the Ultrametric Backbone is included in the Metric Backbone. In hierarchical terms, the relationships can be summarized as follows: PLAM⊂MSP⊂UMB⊂MB

#### High Overlap Above 90%:

High overlap is observed, with, on average, 90% and 97% of the High Salience Skeleton edges overlapping with the Planar Maximally Filtered Graph and Metric Backbones, respectively. On average, 93% of the Primary Linkage Analysis Backbones overlap with the Planar Maximally Filtered Graph Backbone.

#### Overlap Above 80%:

Significant overlap is observed, with, on average, 84% and 86% of the H-Backbone edges overlapping with the Planar Maximally Filtered Graph and Metric Backbones, respectively. On average, 85% of the Ultrametric Backbone edges overlap with the Planar Maximally Filtered Graph Backbone. On average, 83% and 88% of the High Salience Skeleton edges overlap with the edges of the Minimum Spanning Tree and Ultrametric Backbones.

#### Unique Backbones with Doubly Stochastic Filter:

The Doubly Stochastic filter yields unique backbones with low overlap percentages with other methods. The overlap ranges from 8% with the H-Backbone to 45% with the Metric Backbone. In contrast, all methods show a fair overlap, with approximately 55% of their edges overlapping with the Doubly Stochastic Backbones.

These findings underscore the distinctive characteristics and hierarchical relationships of various backbone filtering techniques. The Doubly Stochastic filter generates unique backbones with limited overlap with other methods.

## 5 Experiment 2: Exploring relationships between backbone edges and edge properties

In this experiment, we investigate the relationships between backbone edges and edge properties to understand whether specific properties persist or disappear during filtering. We calculate the Point Biserial correlation between backbone edges and each network property to analyze these associations, including weight, degree, weighted degree, betweenness, and weighted betweenness. The findings are then presented in violin plots, which comprehensively visualize each method’s distribution of correlation values.

### 5.1 Backbone edges and edge weights

**Fig 4:**
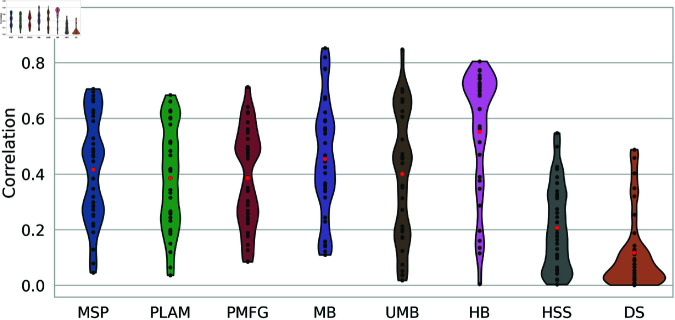
The point biserial correlation distribution between backbone edges and edge weights across all networks is presented as a violin plot. The average correlation is highlighted with a red point for each method.

Fig 4 displays the results of the Point Biserial correlation between backbone edges and edge weights. Analyzing the averages for each method, represented by the red points, reveals that all averages are less than 0.6. This suggests a lack of a strong correlation between the backbone edges and edge weights across all methods.

However, when comparing the methods to each other, two extremes emerge. The Doubly Stochastic method exhibits correlation values highly concentrated at the bottom of the violin plot, with the lowest values and the average being less than 0.2. This indicates that the Doubly Stochastic method is the least sensitive among all methods to edge weights.

In contrast, the H-Backbone correlation values are highly concentrated at the top of the violin plot, with values among the highest and an average approaching 0.6. This implies that the H-Backbone method is more sensitive to edge weights than other methods, suggesting that edges with higher weights are more likely to be included in the backbone.

Between these two extremes, the other methods show no consistent behavior across all networks. The correlation values span between 0 and 0.8, indicating a nuanced relationship influenced by other properties that affect the association between backbone edges and edge weights.

### 5.2 Backbone edges and other edge properties

The Degree of an edge connecting node *i* with node *j* is defined as the product of the degrees of the connected nodes, represented as $k(i,j)=k_{i}\cdot k_{j}$ [100]. High-degree edges often link network hubs, a common feature in scale-free networks. Examining the relationship between edge backbones and edge degree provides insights into how backbone filtering techniques handle hub-connected edges.

Edge betweenness is a measure that quantifies the shortest paths traversing an edge in a network [101]. An edge with a high betweenness score is pivotal in connecting different network parts. Removing such edges can profoundly impact communication efficiency between various nodes by disrupting the shortest link paths.

**Fig 5:**
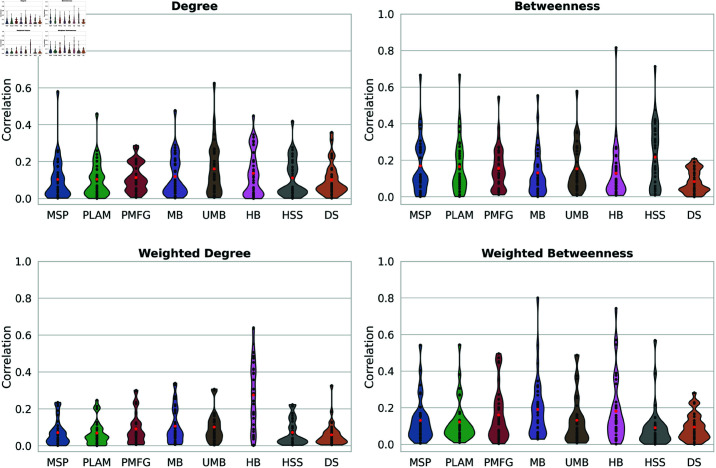
Violin plots illustrating the distribution of point biserial correlation values between backbone edges and various edge properties across all networks. Red points indicate each method’s average point biserial correlation, with edge properties including weight, degree, weighted degree, betweenness, and weighted betweenness under examination.

Both properties introduce a weighted version by considering edge weights. Fig 5 displays the results of the Point Biserial correlation between backbone edges and edge degrees, betweenness, and the weighted versions of these two properties. In the top two panels, we observe that the average correlation is less than 0.25 in all methods for both edge degrees and betweenness. This indicates no strong relation between backbone edges and edge degree or edge betweenness. It suggests that these properties do not significantly influence the edge selection process, regardless of the filtering technique. Similar results are observed for the weighted versions of degree and betweenness in the bottom two panels of Fig 5.

## 6 Experiment 3: Exploring the global properties of the backbones

This section delves into the impact of various methods on shaping the global network features of the extracted backbones. The procedure begins with the extraction of the backbone for each network. Subsequently, the properties of the extracted backbones are computed and normalized. The outcomes are then presented in a violin plot for each backbone extraction method.

### 6.1 Edge fraction

**Fig 6:**
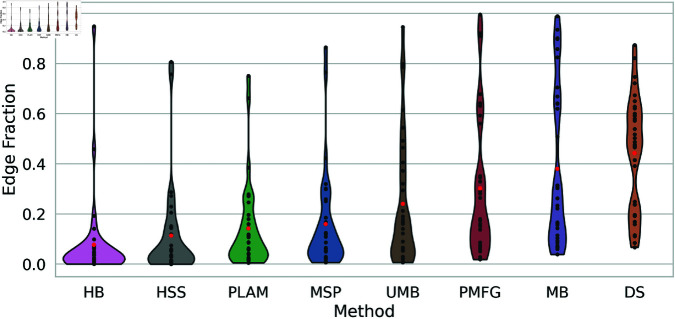
The preserved edge fraction distribution of backbones obtained using diverse filtering techniques across multiple networks presented as a violin plot. Average edge fractions are highlighted with red points for each method.

The primary goal of backbone extraction methods is to diminish the size of a network. As a result, larger networks allow the filtering of more edges than smaller networks. To scrutinize the behavior of these backbone extraction methods. Fig 6 illustrates the edge fraction of backbones obtained through diverse filtering techniques across all networks. This visual representation provides insights into how the methods perform regarding edge reduction across all networks.

The edge fraction distribution of H-backbones is prominently concentrated towards the bottom, with an average of 0.1. This observation underscores the efficacy of the H-backbone method in removing approximately 90% of the edges while preserving around 10%, with a few outliers.

In comparison, the High Salience Skeleton, Primary Linkage Analysis Method, and Minimum Spanning Tree exhibit a similar behavior but with a higher average edge fraction ranging between 0.1 and 0.2. Conversely, the plots of the other methods are more horizontally distributed, indicating a lack of consistent behavior across all networks. Some networks exhibit edge preservation of less than 10%, while others retain more than 50

Notably, the Doubly Stochastic method does not display extreme edge fractions exceeding 0.9 or falling below 0.1, suggesting a more balanced and moderate edge preservation across the networks it processes.

### 6.2 Node fraction

Preserving network nodes throughout the filtering process poses a considerable challenge for backbone filtering techniques. An effective technique should filter network edges without isolating nodes, ensuring the integrity of the network. It should retain the highest proportion of nodes within the network after the edge filtering process.

**Fig 7:**
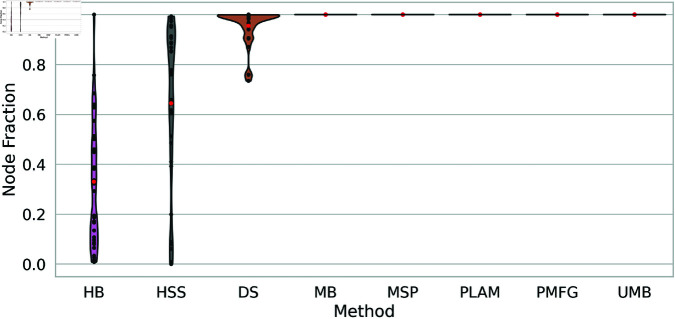
The preserved node fraction distribution of backbones obtained using diverse filtering techniques across multiple networks presented as a violin plot. Average node fractions are highlighted with red points for each method.

Fig 7 depicts the node fraction of backbones obtained through various filtering techniques across all networks. Notably, the Metric Backbone, Ultrametric Backbone, Minimum Spanning Tree, Planar Maximally Filtered Graph Method, and Primary Linkage Analysis filters consistently maintain all nodes across networks of different sizes.

However, the Doubly Stochastic filter is less conservative in preserving the original network’s nodes, with values sometimes falling below 1, indicating node isolation in certain networks. Conversely, the High Salience Skeleton and H-Backbone filters tend to isolate many nodes, with average node fraction values around 0.6 and 0.4, respectively. Interestingly, node fraction values for the High Salience Skeleton are concentrated at the top, indicating a slightly conservative behavior. In contrast, in the H-Backbone, they are concentrated at the bottom, suggesting a more isolating behavior.

### 6.3 Weight fraction

Backbone filtering techniques collectively strive to maintain the diversity of edge weights while filtering network edges. However, our previous experiments have revealed that certain methods show a stronger tendency to preserve high edge weights. This leads us to compare the fractions of retained edge weights by these methods.

**Fig 8:**
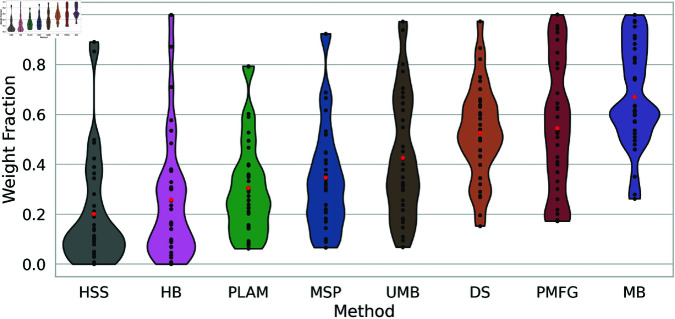
The preserved weight fraction distribution of backbones obtained using diverse filtering techniques across multiple networks presented as a violin plot. Average weight fractions are highlighted with red points for each method.

Fig 8 illustrates the weight fraction of backbones obtained through various filtering techniques across all networks. Notably, only the H-Backbone and High Salience Skeleton preserve a very low weight fraction, typically less than 10%

Conversely, the average weight fraction is around 0.5 for the Doubly Stochastic filter, with weight fractions spanning around the average and a few outliers. The Planar Maximally Filtered Graph Method presents a stick-shaped plot. It indicates a network-dependent behavior that preserves a high weight fraction in some networks while maintaining a minimum weight fraction of 0.2 in others. Lastly, the Metric Backbone boasts the highest average weight fraction, around 0.65. It consistently preserves a high weight fraction, exceeding 0.8 in many networks, with some outliers preserving less than 0.5.

### 6.4 Weight entropy

All backbone filtering techniques share the common objective of maintaining edge weight diversity while filtering network edges. This motivates us to compare the fraction of retained edge weights by these methods to assess their success in preserving these weight scales.

**Fig 9:**
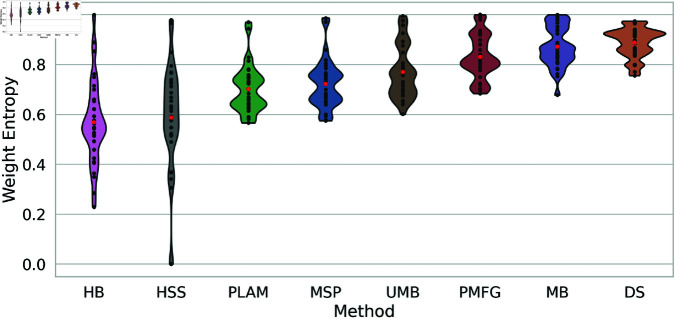
The normalized weight entropy distribution of backbones obtained using diverse filtering techniques across multiple networks presented as a violin plot. Average normalized weight entropy is highlighted with red points for each method.

Fig 9 illustrates the normalized weight entropy of backbones obtained through various filtering techniques across all networks. The H-Backbone and High Salience Skeleton exhibit an average normalized weight entropy of around 0.6 with a high standard deviation. In some networks, the weight entropy is fully preserved, as indicated by a normalized entropy of 1, while in other networks, it decreases to a minimum of approximately 20%.

Conversely, all other methods display higher average normalized weight entropy with low standard deviation. This indicates the effectiveness of these methods in preserving the original network’s weight entropy. The Metric Backbone and Doubly Stochastic backbones are particularly noteworthy, which exhibit very high normalized weight entropy, approximately 0.9. This underscores their ability to maintain the weight entropy across various networks.

### 6.5 Reachability

Reachability measures the connectivity between any pair of nodes within a network. Assessing the reachability of extracted backbones allows us to understand how interconnected these backbones are.

**Fig 10:**
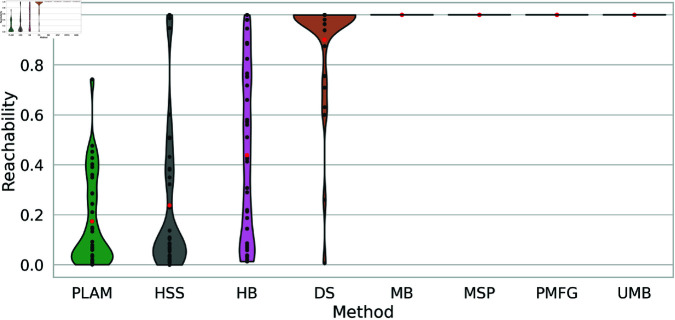
The reachability distribution of backbones obtained using diverse filtering techniques across multiple networks presented as a violin plot. Average reachability is highlighted with red points for each method.

Fig 10 illustrates the reachability of backbones obtained through various filtering techniques across all networks. The Metric Backbone, Planar Maximally Filtered Graph Method, Ultrametric Backbone, and Minimum Spanning Tree Backbones consistently exhibit high reachability across all networks. The Doubly Stochastic backbones also demonstrate high reachability values in most networks, with only a few outliers. These methods showcase their ability to extract connected backbones with high reachability between nodes.

In contrast, the other methods show lower reachability values. The H-Backbone plot resembles a stick, with values between 0 and 1, indicating its network dependence. The High Salience Skeleton and Primary Linkage Analysis Method plots are highly concentrated at the bottom, suggesting low reachability values. Their average reachability is approximately 0.2, indicating a tendency to extract disconnected backbones in most networks.

### 6.6 Number of components

Low reachability values indicate a disconnected network, but they do not provide insight into the specific fragmentation pattern. To better understand the connectedness of the extracted backbones, we investigate the number of components within them.

**Fig 11:**
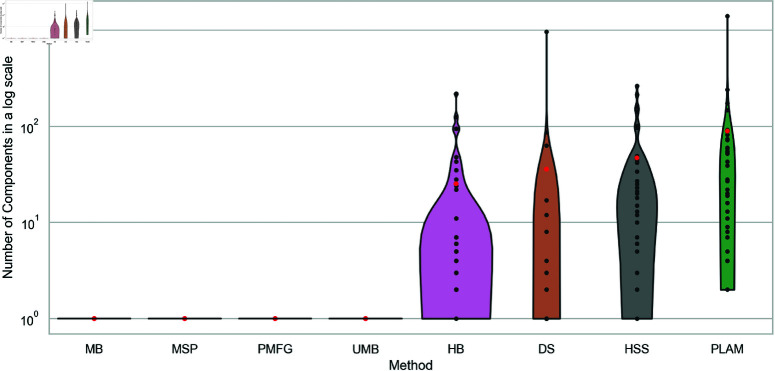
The number of components distribution of backbones obtained using diverse filtering techniques across multiple networks, presented as a violin plot. Average reachability is highlighted with red points for each method. Note, that the y-axis is in log scale.

Fig 11 illustrates the number of components in backbones obtained through various filtering techniques across all networks. Remarkably, the Planar Maximally Filtered Graph Method, Metric Backbone, Ultrametric Backbone, and Minimum Spanning Tree filters consistently exhibit the behavior of extracting a single component.

Conversely, the other methods extract backbones with multiple components. Specifically, the Primary Linkage Analysis filter always retains multiple components. This observation aligns with the low reachability values observed in the previous experiment for these methods, except for the Doubly Stochastic filter. Although this method has many components, it also exhibits high reachability values. This discrepancy can be explained by large connected components in the Doubly Stochastic filter, unlike other methods where the extracted components are smaller.

### 6.7 Transitivity

Transitivity quantifies the likelihood that two neighboring nodes are also connected, offering insights into the interconnectedness among local clusters or triangles within the extracted backbones.

**Fig 12:**
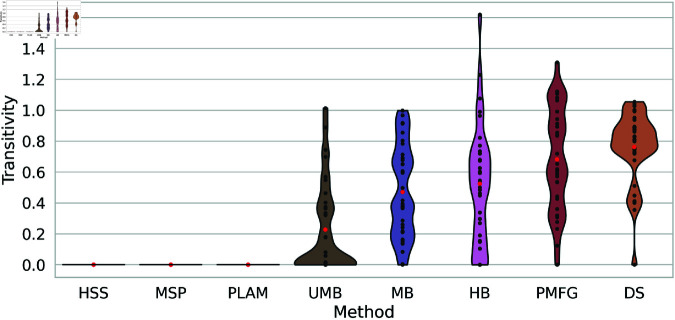
The normalized transitivity distribution of backbones obtained using diverse filtering techniques across multiple networks presented as a violin plot. Average normalized transitivity is highlighted with red points for each method.

Fig 12 illustrates the transitivity of backbones obtained through various filtering techniques across all networks. The Primary Linkage Analysis, Minimum Spanning Tree, and High Salience Skeleton backbones consistently exhibit a transitivity equal to zero, indicating the systematic breaking of all cycles in the network through the filtration process. The Ultrametric Backbone plot shows normalized transitivity concentrated at the bottom, with an average of around 0.2. This method also tends to break many cycles in the network, with some outliers where transitivity values are high and occasionally preserved.

For the Metric Backbone, Planar Maximally Filtered Graph Method, and H-Backbones, there is no consistent behavior across all networks, indicating network dependency. In some networks, cycles are broken, while in others, cycles are less disrupted and more preserved. Notably, the Planar Maximally Filtered Graph Method and H-Backbones sometimes increase transitivity, possibly explained by breaking more network triads instead of cycles.

Finally, the Doubly Stochastic normalized transitivity values are concentrated at the top, with an average of approximately 0.8. This method demonstrates superiority over others in preserving the transitivity of the original network, indicating its efficacy in maintaining local cluster interconnections during the filtering process.

## 7 Experiment 4: Comparing backbone distributions

In the preceding experiment, we delved into the overarching characteristics of backbones and pinpointed attributes linked to each extraction method. We employ a parallel methodology, concentrating on distributions, specifically in weight and degree. The procedure initiates by extracting the backbones for each network utilizing distinct backbone extraction methods. Subsequently, we calculate the Kolmogorov-Smirnov statistic for each backbone distribution compared to the original network. Following this, we depict the KS statistic for each method across all networks in a violin plot. Finally, within each network, methods are ordered based on the KS statistic values, with the ranking reflecting the degree of alignment of each method with the original network. Ultimately, the outcomes are presented in a box plot.

### 7.1 Weight distribution

The comparison of the weight distribution of the filtered network backbone is crucial in evaluating the effectiveness of the filtering technique in retaining different weight scales. The weight scales in a network reflect the connections and relationships between the nodes, and a suitable filtering technique should preserve different scales of these weights, thus preserving the original weight distribution. This comparison allows us to identify which filtering methods best maintain the original network weight distribution.

**Fig 13:**
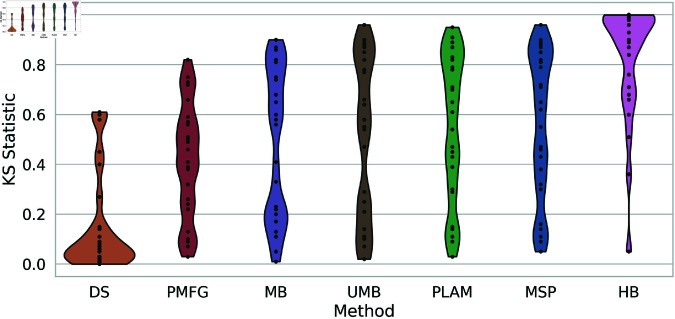
The violin plots of the KS-static values of each backbone extraction method across all networks. The KS-static represents the distance between the backbone and the original weight distribution.

In Fig 13, the violin plots illustrate the KS-statistic values for each backbone extraction method across all networks. Two distinct extreme behaviors are evident. On the extreme left, represented by the Doubly Stochastic (DS), there is a notable concentration of values between 0 and 0.2, with a few outliers creating an upward tail. This signifies the superiority of this method in accurately capturing the original weight distribution within the extracted backbones. Conversely, on the extreme right, depicted by the H-Backbone (HB), there is a high concentration of values between 0.8 and 1, with outliers causing a downward tail in the plot. This indicates that this method consistently falls short of capturing the original weight distribution. Between these extremes, the remaining methods consistently exhibit uniform values across all scales. This characteristic is depicted in the violin plot, resembling sticks. This suggests a network dependency, wherein these methods effectively capture the original weight distribution in specific networks but face challenges in others.

**Fig 14:**
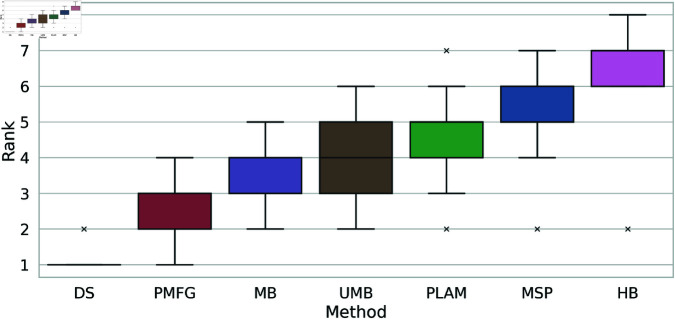
The boxplots of the backbone extraction methods rank based on the Kolmogorov-Smirnov statistic between the cumulative weight distribution of each filtering technique and the original network across all networks.

Thus far, we have identified the most effective and least effective methods for capturing the original weight distribution. To rank the remaining methods, we analyze the boxplots illustrating the rankings of backbone extraction methods based on the Kolmogorov-Smirnov statistic across all networks, as depicted in Fig 14. Clearly, the Doubly Stochastic (DS)and H-Backbone (HB) occupy the first and last ranks, respectively. The Planar Maximally Filtered Graph Method (PMFG) is positioned between them, securing ranks 2 and 3, followed by the Metric backbone (MB), falling between ranks 3 and 4. The Ultrametric Backbone (UMB) attains an average rank of 4, while the Primary Linkage Analysis (PLAM) and Minimum Spanning Tree (MSP) methods rank between (4 and 5) and (5 and 6), respectively.

### 7.2 Degree distribution

Evaluating the degree distribution of the filtered network backbone is pivotal in assessing the filtering technique’s effectiveness in preserving diverse degrees of connectivity. Node degrees signify the connections and influence of nodes in a network, and an effective filtering method should retain various degrees, thereby conserving the original degree distribution. This comparative analysis enables the identification of filtering methods that most effectively uphold the inherent diversity of node degrees in the original network.

**Fig 15:**
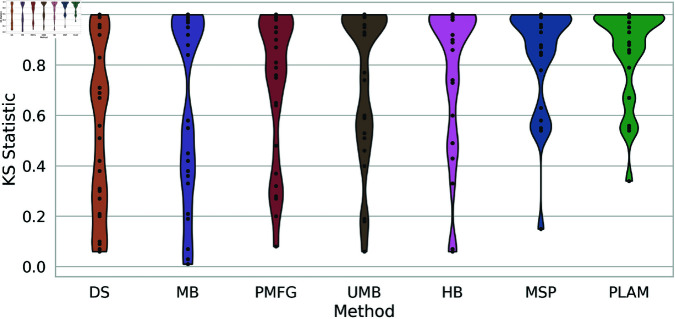
The violin plots of the KS-static values of each backbone extraction method across all networks. The KS-static represents the distance between the backbone and the original degree distribution.

In Fig 15, the violin plots depict the KS-statistic values for each backbone extraction method across all networks, revealing two distinct extreme behaviors. On the extreme left, represented by the Doubly Stochastic (DS), the values are well-distributed between 0 and 1. This suggests a network dependency, where these methods adeptly capture the original degree distribution in specific networks. Conversely, on the extreme right, illustrated by the Primary Linkage Analysis Method (PLAM), there is a high concentration of values between 0.8 and 1, with outliers causing a downward tail in the plot. This indicates that this method consistently falls short of capturing the original degree distribution. Between these extremes, the values of the remaining methods are slightly concentrated at the top with longer tails. This signifies that these methods effectively capture the original degree distribution in specific networks but face challenges in others.

**Fig 16:**
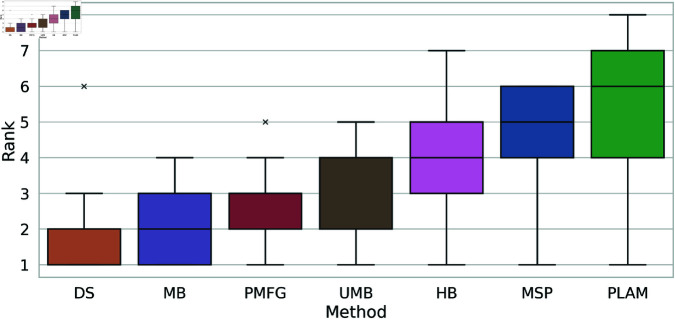
The boxplots of the backbone extraction methods rank based on the Kolmogorov-Smirnov statistic between the cumulative degree distribution of each filtering technique and the original network across all networks.

Up to this point, we have identified the most and least effective methods for capturing the original degree distribution. To rank the remaining methods, we examine the boxplots illustrating the rankings of backbone extraction methods based on the Kolmogorov-Smirnov statistic across all networks, as depicted in Fig 16. The Doubly Stochastic (DS) and Primary Linkage Analysis (PLAM) methods occupy the first and last ranks, respectively. The Metric Backbone (MB) is positioned between them with an average rank of 2, followed by the Planar Maximally Filtered Graph Method (PMFG), falling between ranks 2 and 3. The Ultrametric Backbone (UMB) attains an average rank of 3, while the H-Backbone (HB) and Minimum Spanning Tree (MSP) methods hold average ranks of 4 and 5, respectively.

## 8 Discussion

This study compares eight structural backbone edge filtering techniques across a dataset of 33 networks with various sizes and topological properties from different domains. The primary objectives are to evaluate the similarity between filtering techniques and identify properties associated with each backbone extraction method.

In the initial experiment, the investigation into the similarity between backbone filtering techniques reveals a hierarchical relationship among backbone methods (PLAM⊂MSP⊂UMB⊂MB). The Primary Linkage Analysis Backbone *PLAM* captures the most influential edges at the level of each node. The Minimum Spanning Tree Backbone *MSP* encompasses edges forming a maximum spanning tree, including those from *PLAM*. The Ultrametric Backbone *UMB* focuses on maximum-distance paths, incorporating edges from *MSP*. The Metric Backbone *MB* emphasizes shortest paths based on the sum of edge distances, including edges from *UMB*. These relationships imply that each method adds complexity or specificity to the selected edges, with *PLAM* focusing on influential edges, *MSP* emphasizing overall network connectivity, *UMB* prioritizing paths with maximum distances, and *MB* concentrating on paths with minimum total edge distances. This relation stems from their reliance on similar global approaches, such as shortest paths and connectivity. In contrast, the Doubly Stochastic Filter exhibits distinct behavior. It prioritizes local adjacency relationships through iterative normalization. This leads to minimal overlap with hierarchical methods (e.g., 8% with *H*-Backbone, 45% with *MB*). This contrast shows two complementary approaches. Shortest-path-based methods preserve global network structure, while the Doubly Stochastic Filter emphasizes localized relationships.

Substantial overlap between the High Salience Skeleton with Planar Maximally Filtered Graph and Metric Backbones indicates that the high salience skeleton backbone is nearly planar, with only a few edges breaking this planar property. Since the Metric backbone is unaffected by the reduction of the original graph, no shortest path is impacted. The High Salience Skeleton filter preserves over 90% of these shortest paths and removes only a few due to threshold settings.

The pronounced overlap between Primary Linkage Analysis and Planar Maximally Filtered Graph is expected, as both methods prioritize high-weight edges. However, the former operates at the node level, while the latter considers the entire graph. Similarly, the H-Backbone filters overlap with the Planar Maximally Filtered Graph and Metric Backbones.

In the second experiment, the focus shifts to the relationships between backbone edges and local edge properties. Examining edge weight, edge degree, edge betweenness, and their weighted versions reveals inconsistent relations between backbone edges and edge weights across networks. However, it identifies that high-weight edges are more likely present in the H-Backbone and less likely in the Doubly Stochastic and High Salience Skeleton Backbones. Other methods display inconsistent behaviors, indicating network-dependent relations affected by other network properties. No correlation is found between backbone edges and other edge properties across all networks, suggesting these properties do not influence the filtering process.

**Table 2:** A recap of the characteristics of backbone filtering techniques. The symbol ✓ signifies adherence to the property, ▲ denotes network dependency, and ✖ indicates non-conformance to the property.

Property	MSP	DS	HSS	HB	MB	UMB	PLAM	PMFG
Heavy Filtering	✓	▲	✓	✓	▲	▲	✓	▲
Node Preservative	✓	▲	▲	✖	✓	✓	✓	✓
Entropy Preservative	✓	✓	▲	▲	✓	✓	✓	✓
Connectivity Preservative	✓	✓	✖	▲	✓	✓	✖	✓
Transitivity Preservative	✖	✓	✖	▲	▲	✖	✖	▲

The third experiment involves extracting backbones and analyzing their global topological properties, such as the fraction of preserved edges, nodes, weights, weight entropy, reachability, number of components, and transitivity. Results, presented in Table 2, highlight consistent preservative behaviors in green, network-dependent behaviors in blue, and consistent non-preservative behaviors in red.

For example, the Minimum Spanning Tree filter significantly reduces graph density through heavy filtering while preserving other properties and breaking cycles. The Primary Linkage Analysis filter exhibits similar heavy filtering behavior but disrupts network connectivity during filtration.

The H-Backbone filter consistently demonstrates heavy filtering behavior but fails to preserve network nodes, with other properties depending on the specific network.

The High Salience Skeleton filter consistently exhibits heavy filtering behavior but fails to preserve network connectivity and transitivity, while node preservation and weight entropy depend on the specific network.

The Metric Backbone and Planar Maximally Filtered Graph filters consistently preserve all nodes in the backbone, maintaining their connectivity and weight entropy. The density of filtration and transitivity are network-dependent. The Ultrametric Backbone filter behaves similarly but consistently breaks cycles and disturbs network transitivity.

The Doubly Stochastic filter consistently preserves weight entropy, network connectivity, and transitivity. However, graph density and node preservation are network-dependent.

Furthermore, we investigate the influence of the network categories on the backbone properties. To do so, for each property under investigation, we plot a heatmap with the backbone methods on the x-axis and the network categories on the y-axis. The figures are provided in the Supporting information. Results show that the network type does not influence the performance and properties of the filtering techniques.

Finally, examining the weight and degree distributions of the extracted backbones reveals that the Doubly Stochastic filter better captures the weight distribution of the original network. In contrast, the H-Backbone filter exhibits the most considerable deviation. Regarding degree distribution, the Doubly Stochastic and Metric Backbone filters better preserve it than other methods, with the Primary Linkage Analysis and Minimum Spanning Tree filter’s backbones deviating the most.

Overall, these comprehensive experiments provide valuable insights into the behavior and characteristics of different structural backbone filtering techniques, shedding light on their strengths, weaknesses, and unique features in diverse network contexts.

## 9 Conclusion

In conclusion, this comprehensive study has systematically compared eight structural backbone edge filtering techniques across a diverse set of 33 networks spanning various sizes, topological properties, and domains. The goal was to assist users in selecting the most suitable technique for extracting a network backbone, a process crucial for expediting analysis and enhancing visualization in diverse applications.

The overlap analysis uncovered distinctive behavior in the Doubly Stochastic Filter, and hierarchical relationships among the backbones (PLAM⊂MSP⊂UMB⊂MB) highlighted the incremental complexity added by each subsequent method. The second experiment revealed significant overlaps between certain filtering techniques, such as the High Salience Skeleton with Planar Maximally Filtered Graph and Metric Backbones, indicating planarity and specific edge preservation characteristics.

The correlation analysis identifies a tendency for high-weight edges to be more prevalent in the H-Backbone and less likely in the Doubly Stochastic and High Salience Skeleton Backbones. Conversely, other filtering methods exhibit inconsistent behaviors, indicating that other network properties influence these relationships and are, thus, network-dependent.

The properties analysis conducted in this study revealed three distinctive behavioral types for each topological property: consistent, inconsistent, and network-dependent behaviors, as succinctly summarized in Table 2. Notable observations include the Doubly Stochastic filter as the singular method effectively preserving network transitivity. Conversely, the Minimum Spanning Tree, High Salience Skeleton, H-Backbone, and Primary Linkage Analysis filters significantly reduced network size. Among the filters, the Minimum Spanning Tree, Doubly Stochastic, Metric, Ultrametric Backbone, and Planar Maximally Filtered Graph demonstrated efficacy in preserving network connectivity and weight entropy.

Distribution analysis brought to light the unique proficiency of the Doubly Stochastic filter, setting it apart from all other methods, particularly in capturing the original weight and degree distributions. The Metric Backbone filter closely followed suit, showcasing similar capabilities in weight distribution.

These findings provide valuable insights for users who select an appropriate backbone extraction method based on individual method properties. It is worth noting that future research endeavors will expand on this investigation, encompassing additional backbone extraction methods and properties further to enhance our understanding of network backbone extraction techniques. The systematic comparison presented here lays a solid foundation for informed decision-making in choosing the most suitable method for a given analytical or visualization task in diverse network applications.

## Supporting information

S1 File Provides a detailed description of the Dataset. (PDF)

S2 File Provides a detailed description for the Network Properties. (PDF)

S3 File Provides a detailed description for the Evaluation Measures. (PDF)

S4 File Provides detailed results of the influence of network categories on the backbone properties. (PDF)
