# Supplementary material for: Exploring weighted network backbone extraction: A comparative analysis of structural techniques
Source: PLoS One. 2025 May 20;20(5):e0322298. doi: 10.1371/journal.pone.0322298 (PMC12091788; doi:10.1371/journal.pone.0322298)
Supplement: S1 File — (PDF) [file pone.0322298.s001.pdf]

1 Dataset Overview

The experiments involve 33 real-world networks spanning character, web, biological, economic, infrastructural, and offline/online social networks. Table 1 reports their basic topological features.

1.1 Character networks:

In the *Les Misérables* network [1], nodes represent actors in Victor Hugo's novel and are connected if they appear in the same chapter of the Les Misérables novel. Edge weights denote the number of such occurrences.

In the *Game of Thrones* network [2], nodes represent the characters in the Game of Thrones book ”A Storm of Swords”, and edges represent their co-appearances if they appeared within 15 words in a given text. Edge weights denote the number of such occurrences.

1.2 Web networks:

In the *Webkb Cornell* and *Webkb Washington* networks [3], nodes represent projects, and student web pages crawled from the Cornell and Washington Science departments in 1998, respectively. An edge exists if both nodes point to the same page through a hyperlink. Edge weights count the number of hyperlinks.

1.3 Biological networks:

The *Budapest Connectome* network [4, 5] is a parameterizable consensus brain graph derived from connectomes of 477 people, each computed from MRI datasets of the Human Connectome Project. Nodes are brain regions, and edges are ”tracks” that run between two regions. Weights are the number of occurrences in each of the 477 individuals.

In the *Human Connectome* network [6] nodes represent brain regions and edges are pathways mapped using DSI (diffusion spectrum imaging) of a group of five human participants in a study to map the human brain. Edge weights are the average volume of cortico-cortical axonal pathways between the human brain regions of the five participants.

The *C. elegans* network [7] is the corrected version of the neuron network of adult hermaphrodite worms C. elegans, constructed from electron microscopy series, to include edges(gap junction). Edge weights correspond to the total number of EM serial sections of connectivity.

The *Messel Shale* [8] is a food web network of feeling links among taxa based on the 48 million years old uppermost early Eocene Messel Shale. Edge weight denotes the certainty of the edge.

In the *Florida Bay* network [9], nodes are species, and edges denote the flow of carbon between species, typically via predation but not necessarily so. Edge weights correspond to the amount of carbon transferred.

1.4 Economic networks:

The *Faculty Hiring US* [10] is a network of faculty hiring for all PhD-granting US universities for all of the academia over the decade 2011–2020. Each node is a PhD-granting institution, and a directed edge indicates that a person received their Ph.D. from an institution and was tenure-track faculty at another institution during collection. Edge weight corresponds to the total count of hires of men and women.

In the *Job Mobility* network `https://www.michelecoscia.com/?page_id=312`, nodes are occupations in the US derived from the CensusBureau's Current Population Survey `https://www.census.gov/programs-surveys/cps.html`. Edge weights are the number of workers who changed jobs from one occupation in 2009 to another in 2010.

1.5 Infrastructural networks:

In The *Worldwide Air Transportation* [11,12] and *Openflights* [13] nodes are world airports. The former edges are direct flights connecting two airports, and edge weights represent the flights offered by different companies between 17 May and 22 May 2018. While for the latter edges are routes between two non-US-based airports downloaded from Openflights `https://openflights.org` on August 12, 2011, and edge weights represent the number of routes between two airports.

In the *US Airports 500* network [14], nodes represent the top 500 busiest commercial airports in the United States. An edge exists between two airports if a flight was scheduled between them in 2002. The weights correspond to the number of seats available on the scheduled flights.

In *Paris Bus* and *Paris Rail* [15] nodes represent bus and rail stops. An edge exists if a route connects two stops. The weights correspond to the number of vehicles that have traveled between two stops within a time interval.

In the *GLSN 2015* [16] nodes represents international ports. An edge exists if two ports are connected by the same service route from a dataset of 1316 international liner shipping service routes in 2015. Edge weights represent total traffic capacity (measured in TEU).

1.6 Offline social networks:

The *Davis' Southern Women Club* [17] was collected by Davis and colleagues in the 1930s. Nodes represent 18 Southern women. An edge exists if two women attend at least one social event. Edge weights are the number of co-attendances at 14 social events.

In the *Karate Club* network [18], nodes are individuals from Zachary's karate club. An edge exists between two nodes if the corresponding individuals are seen together outside the normal club activities. Edge weights represent the number of such occurrences.

In the *Madrid Train Bombing Terrorists* network [19], nodes represent terrorists involved in the 2004 Madrid train bombing. Edges are associations among them, and edge weights denote how 'strong' a connection was. This includes friendship and co-participating in training camps or previous attacks.

In the *Illinois High School Students* network [20], nodes are male students in a small high school in Illinois from 1958. An edge exists if an individual names another as a friend in either of two identical surveys (from the Fall and Spring semesters). Edge weights are the number of surveys in which the friendship was named.

In the *ANU Residence Hall* network [21], nodes are students living in a residence hall at Australian National University (date unknown). An edge exists if a resident names another as a friend and edge weight indicates the level of friendship: 5 (best

friend), 4 (close friend), 3 (friend), 2, 1.

In the *Windsurfers* network [22], nodes represent windsurfers in southern California during the Fall of 1986. Edges represent interpersonal contacts among the windsurfers, and edge weights indicate the perception of social affiliations majored by the tasks in which each individual was asked to sort cards with other surfer's names in the order of closeness.

In the *American High School* network (US-HS) [23] nodes represent students, teachers, and staff from an American High School. An edge exists if two individuals met with a time resolution of approximately 20 seconds on January 14th, 2010. Edge weights correspond to the sum of contact pairs.

In the *American Middle School* network (US-MS) [24] nodes represent students of 7th and 8th grades in a suburban middle school in Utah (USA). Edge weights correspond to the sum of contact pairs on November 28th and 29th, 2012.

In the *American Elementary School* (US-ES) [24] nodes represent students of Kindergarten grades (K-6) in a suburban elementary school in Utah (USA). Edge weights correspond to the sum of contact pairs during January 31th and February 2nd, 2013.

In the *French Primary School* network(Fr-PS) [25] nodes represent (232 students and 10 teachers) in a primary school in Lyon, France. Edge weights correspond to the number of face-to-face contacts recorded using active RFID devices on October 1st and 2nd, 2009.

In the *French High School* network (Fr-HS) [25] nodes represent 327 high school students of specific classes called "classes préparatoires" in Lycée Thiers, Marseille, France. Edge weights correspond to the contacts between students over 4 days in 2013.

In the *Workplace* network (Fr-Wo) [25] nodes represent 232 individuals working in one of the two office buildings of the French Institute for Public Health Surveillance (Institut de veille sanitaire; InVS), located near Paris, France. Edge weights correspond to the number of contacts between individuals over 12 days in 2015.

In the *ACM Hypertext 2009 Scientific Conference* network (It-SC) [25] nodes represent 113 individuals attending the ACM Hypertext 2009 Conference in Torino, Italy. Edge weights correspond to the number of contacts between individuals for 3 days in 2009.

In the *Geriatric Ward of French Hospital* network (Fr-Ho) [25] nodes represent 46 hospital employees and 29 patients (75 total individuals) in a short-stay geriatric unit (19 beds) of a university hospital of almost 1,000 beds, located in Lyon, France. Edge weights correspond to the number of contacts between individuals for 4 days, from December 6th to the 10th, 2010.

1.7 Online social networks:

The *Facebook Like Forum* [26] is a projection of an interesting two-mode network among users and topics in which a weight can be assigned to the ties based on the number of messages that a user posted on a topic. The projection is weighted by the sum of messages that both users post on a topic.

In the *Sports Cotagging* network [27], nodes represent tags from the Sports Stack-Exchange questions `https://sports.stackexchange.com/`. An edge exists if two tags were co-tagged in a question, and edge weights indicate the number of times of co-tagging.

Table 1. Topological features of the networks. N is the number of nodes. $|E|$ is the number of edges. $< k >$ is the average degree. ρ is the density, a is the assortativity, d is the diameter, and c is the average clustering coefficient.

Type	Network	N	$\|E\|$	$< k >$	ρ	a	d	c
Biological	Budapest Connectome 3	1015	120748	237.927	0.235	0.005	4	0.727
	Human Connectome	998	41693	83.553	0.084	0.153	4	0.419
	Messel Shale	700	6395	18.271	0.026	-0.165	6	0.104
	Florida Bay	128	2075	32.422	0.255	-0.112	3	0.335
	C. elegans	460	1432	6.226	0.014	0.131	34	0.194
Offline Social	US-HS	788	118291	300.231	0.381	0.054	3	0.499
	US-MS	591	56867	192.443	0.326	0.011	2	0.39
	US-ES	339	16546	97.617	0.289	0.13	3	0.452
	Fr-PS	242	8317	68.736	0.285	0.118	3	0.526
	Fr-HS	327	5818	35.584	0.109	0.033	4	0.504
	Fr-Wo	217	4274	39.392	0.182	0.044	5	0.381
	It-SC	113	2196	38.867	0.347	-0.123	3	0.535
	ANU Residence Hall	217	1839	16.949	0.078	0.096	4	0.363
	Fr-Ho	75	1139	30.373	0.41	-0.181	3	0.64
	Windsurfers	43	336	15.628	0.372	-0.147	3	0.653
	Illinois High School	70	274	7.829	0.113	0.083	6	0.465
	Madrid Train Bombing	64	243	7.594	0.121	0.029	6	0.622
	Southern Women Club	18	139	15.444	0.908	-0.176	2	0.937
	Karate Club	34	78	4.588	0.139	-0.476	5	0.571
Economical	Faculty Hiring US	3284	51805	31.55	0.01	-0.362	5	0.485
	Job Mobility	503	26448	105.161	0.209	-0.245	4	0.636
Infrastructural	Worldwide Air Transportation	2734	16665	12.191	0.004	-0.047	12	0.464
	Openflights	2905	15645	10.771	0.004	0.049	14	0.456
	Paris Bus	10644	12309	2.313	0.0	0.027	159	0.005
	GLSN 2015	777	12000	30.888	0.04	-0.027	5	0.722
	US Airports 500	500	2980	11.92	0.024	-0.268	7	0.617
	Paris Rail	313	408	2.607	0.008	0.353	28	0.117
Web	Webkb Washington	434	15231	70.189	0.162	0.245	4	0.811
	Webkb Cornell	346	13416	77.549	0.225	0.349	4	0.758
Online Social	Facebook Like Forum	869	9999	23.013	0.027	-0.701	6	0.0
	Sports Cotagging	237	1900	16.034	0.068	-0.288	4	0.63
Character	Game of Thrones	107	352	6.579	0.062	-0.131	6	0.551
	Les Misérables	77	254	6.597	0.087	-0.165	5	0.573

References

1. Knuth D. The Stanford GraphBase. A platform for combinatorial computing; 1993.

2. Beveridge A, Shan J. Network of Thrones. Math Horizons. 2016;23:18. doi:10.4169/mathhorizons.23.4.18.

3. Slattery S, Craven M. Combining statistical and relational methods for learning in hypertext domains. vol. 1446; 1998.

4. Szalkai B, Kerepesi C, Varga B, Grolmusz V. The Budapest Reference Connectome Server v2.0. Neuroscience Letters. 2015;595. doi:10.1016/j.neulet.2015.03.071.

5. Szalkai B, Kerepesi C, Varga B, Grolmusz V. Parameterizable consensus connectomes from the Human Connectome Project: the Budapest Reference Connectome Server v3.0. Cognitive Neurodynamics. 2017;11. doi:10.1007/s11571-016-9407-z.

6. Hagmann P, Cammoun L, Gigandet X, Meuli R, Honey C, Wedeen V, et al. Mapping the Structural Core of Human Cerebral Cortex. PLoS biology. 2008;6:e159. doi:10.1371/journal.pbio.0060159.

7. Cook SJ, Jarrell TA, Brittin CA, Wang Y, Bloniarz AE, Yakovlev MA, et al. Whole-animal connectomes of both Caenorhabditis elegans sexes. Nature. 2019;571(7763):63–71.

8. Dunne J, Labandeira C, Williams R. Highly resolved early Eocene food webs show development of modern trophic structure after the end-Cretaceous extinction. Proceedings Biological sciences / The Royal Society. 2014;281:20133280. doi:10.1098/rspb.2013.3280.

9. Ulanowicz R, Heymans J, Egnotovich M. Network Analysis of Trophic Dynamics in South Florida Ecosystems, FY 99: The Graminoid Ecosystem. FY97: The Florida Bay Ecosystem. 2000;.

10. Wapman K, Clauset A, Larremore D. Quantifying hierarchy and dynamics in US faculty hiring and retention. Nature. 2022;610:1–8. doi:10.1038/s41586-022-05222-x.

11. Alves L, Aleta A, Rodrigues F, Moreno Y, Amaral L. Centrality anomalies in complex networks as a result of model over-simplification. New Journal of Physics. 2020;22. doi:10.1088/1367-2630/ab687c.

12. Diop IM, Cherifi C, Diallo C, Cherifi H. Revealing the component structure of the world air transportation network. Applied Network Science. 2021;6(1):1–50.

13. Opsahl T. Why Anchorage is not (that) important: Binary ties and Sample selection; 2011. Available from: `https://wp.me/poFcY-Vw`.

14. Colizza V, Pastor-Satorras R, Vespignani A. Reaction-diffusion processes and metapopulation models in heterogeneous network. Nature Phys. 2007;3. doi:10.1038/nphys560.

15. Kujala R, Weckström C, Darst R, Mladenovic M, Saramäki J. A collection of public transport network data sets for 25 cities. Scientific Data. 2018;5:180089. doi:10.1038/sdata.2018.89.

16. Xu M, Pan Q, Xia H, Masuda N. Estimating international trade status of countries from global liner shipping networks. Royal Society Open Science. 2020;7:200386. doi:10.1098/rsos.200386.

17. Clark TD, Davis A, Gardner BB, Gardner MR, Warner WL. Deep South: A Social Anthropological Study of Caste and Class. The Journal of Southern History. 1942;8. doi:10.2307/2191429.

18. Shore KA. Complex networks: principles, methods and applications. Contemporary Physics. 2018;doi:10.1080/00107514.2018.1450296.

19. Hayes B. Connecting the Dots: Can the tools of graph theory and social-network studies unravel the next big plot? American Scientist. 2006;94:400.

20. Coleman JS. Introduction to Mathematical Sociology.; 1964. Available from: https://www.abebooks.com/Introduction-Mathematical-Sociology-COLEMAN-James-S/189127582/bd.

21. Freeman LC, Webster CM, Kirke DM. Exploring social structure using dynamic three-dimensional color images. Social Networks. 1998;20:109–118. doi:10.1016/S0378-8733(97)00016-6.

22. Freeman L, Freeman S, Michaelson A. On human social intelligence. Journal of Social and Biological Systems. 1988;11:415–425. doi:10.1016/0140-1750(88)90080-2.

23. Stehlé J, Voirin N, Barrat A, Cattuto C, Colizza V, Isella L, et al. Simulation of an SEIR infectious disease model on the dynamic contact network of conference attendees. BMC medicine. 2011;9:87. doi:10.1186/1741-7015-9-87.

24. Toth D, Leecaster M, Pettey W, Gundlapalli A, Gao H, Rainey J, et al. The role of heterogeneity in contact timing and duration in network models of influenza spread in schools. Journal of the Royal Society, Interface / the Royal Society. 2015;12. doi:10.1098/rsif.2015.0279.

25. SocioPatterns: data-driven social dynamics and human activity.;. Available from: http://www.sociopatterns.org.

26. Opsahl T. Triadic closure in two-mode networks: Redefining the global and local clustering coefficients. Social Networks. 2013;35. doi:10.1016/j.socnet.2011.07.001.

27. Fu X, Yu S, Benson AR. Modelling and analysis of tagging networks in Stack Exchange communities. Journal of Complex Networks. 2020;8.
