# Supplementary material for: Exploring weighted network backbone extraction: A comparative analysis of structural techniques
Source: PLoS One. 2025 May 20;20(5):e0322298. doi: 10.1371/journal.pone.0322298 (PMC12091788; doi:10.1371/journal.pone.0322298)
Supplement: S2 File — (PDF) [file pone.0322298.s002.pdf]

1 Network properties

This subsection defines the used network properties in the evaluation process.

1.1 Edge Degree

The Degree of an edge [1] connecting node i with node j can be defined as the product of the degrees of the incident nodes:

$$k(i,j) = k_i k_j \tag{1}$$

Thus edges associated with high degrees connect high-degree nodes in the network (hubs).

1.2 Edge Betweenness

The Betweenness [2] of an edge e is the sum of the fraction of all-pairs shortest paths that pass through the edge e. This follows:

$$b(e) = \sum_{s,t \in N} \frac{\sigma(s,t|e)}{\sigma(s,t)} \tag{2}$$

where N is the set of nodes, $\sigma(s,t)$ is the number of shortest $(s,t)-$paths, and $\sigma(s,t|e)$ is the number of those paths passing through edge e.

1.3 Reachability

The Reachability [3] quantifies the connectivity between any pair of nodes in a network. It is defined as the fraction of node pairs that can communicate with each other. This reads:

$$R = \frac{1}{n(n-1)} \sum_{i \neq j \in G} R_{ij}. \tag{3}$$

with n is the number of nodes and $R_{ij} = 1$ if path exists between node i and j and $R_{ij} = 0$ otherwise. The Reachability values are in the $[0,1]$ range. If any pair of nodes can communicate in a network, the reachability R becomes 1. If $R = 0$ it means all nodes are isolated from each other.

1.4 Transitivity

The transitivity [4] of a network reflects how likely neighboring nodes are connected. Mathematically, it's computed as the ratio of the number of triangles (Δ) to the number of connected triples of nodes (τ):

$$T = \frac{3 \times \Delta}{\tau} \tag{4}$$

1.5 Weight Entropy

Shannon entropy [5] measures the average uncertainty or randomness in a probability distribution. It is calculated using the equation:

$$H(X) = -\sum_{i} P(x_i) \log_2(P(x_i)) \tag{5}$$

where $P(x_i)$ represents the probability of the i-th outcome of the random variable X. Here we define the weight entropy as the Shannon Entropy for the weight distributions.

References

1. Oehlers M, Fabian B. Graph metrics for network robustness—a survey. Mathematics. 2021;9. doi:10.3390/math9080895.

2. Brandes U. On variants of shortest-path betweenness centrality and their generic computation. Social networks. 2008;30(2):136–145.

3. Sato Y, Ata S, Oka I. A strategic approach for re-organization of internet topology for improving both efficiency and attack tolerance; 2008. p. 331 – 338.

4. Wasserman S, Faust K. Social network analysis: Methods and applications. 1994;.

5. Shannon CE. A mathematical theory of communication. The Bell system technical journal. 1948;27(3):379–423.
