# Supplementary material for: Exploring weighted network backbone extraction: A comparative analysis of structural techniques
Source: PLoS One. 2025 May 20;20(5):e0322298. doi: 10.1371/journal.pone.0322298 (PMC12091788; doi:10.1371/journal.pone.0322298)
Supplement: S3 File — (PDF) [file pone.0322298.s003.pdf]

1 Evaluation measures

This subsection defines the tools and measures used in the experiments.

1.1 Point Biserial Correlation

The Point Biserial correlation coefficient [1] measures the strength and direction of the relationship between a binary (dichotomous) variable and a continuous variable. To calculate r_pb, assume that the dichotomous variable Y has the values 0 and 1. Suppose we divide the data set into two groups: group 1, which received the value "1" on Y, and group 2, which received the value "0" on Y. In that case, the point-biserial correlation coefficient is calculated as follows:

$$r = \frac{M_1 - M_0}{s_n} \sqrt{\frac{n_1 n_0}{n^2}} \tag{1}$$

where s_n is the standard deviation used when data are available for every member of the population: $s_n = \sqrt{\frac{1}{n} \sum_{i=1}^{n} (X_i - \bar{X})^2}$. M_1 is the mean value of the continuous variable X for all data points in group 1, and M_0 is the mean value of the continuous variable X for all data points in group 2. Further, n_1 is the number of data points in group 1, n_0 is the number of data points in group 2, and n is the total sample size.

1.2 Jaccard score

The Jaccard score [2] quantifies the similarity between two sets, A and B, by computing the ratio between the intersection's cardinality and the union's cardinality. This reads:

$$J = \frac{|A \cap B|}{|A \cup B|} \tag{2}$$

The score ranges between 0 and 1. A value $J = 1$ indicates that A and B are the same set, while a value $J = 0$ denotes that the sets are completely different.

1.3 Overlap Coefficient

The overlap coefficient [3] or Szymkiewicz–Simpson coefficient is a similarity measure that measures the overlap between two finite sets. It is related to the Jaccard index and is defined as the size of the intersection divided by the smaller of the size of the two sets:

$$overlap(X, Y) = \frac{|X \cap Y|}{min(|X|, |Y|)} \tag{3}$$

However, in the context of this study, we calculate the overlap between the two sets based on the perspective of each set, following the formula:

$$overlap(X, Y)_X = \frac{|X \cap Y|}{|X|} \tag{4}$$

1.4 Two-Sample Kolmogorov-Smirnov

The two-sample Kolmogorov-Smirnov (KS) test tests whether two samples follow the same distribution. [4]. Simply put, the KS statistic for the 2-sample test is the greatest distance between each sample's CDFs (Cumulative Distribution Function). Thus, the Kolmogorov-Smirnov statistic D is given by:

$$D_{m,n} = \max_{x} |F(x) - G(x)| \tag{5}$$

where $F(x)$ and $G(x)$ represent the CDF of the two samples, and n and m are the numbers of observations of the first and second samples, respectively.

References

1. MacCallum RC, Zhang S, Preacher KJ, Rucker DD. On the practice of dichotomization of quantitative variables. Psychological Methods. 2002;7(1):19–40. doi:10.1037/1082-989x.7.1.19.

2. Jaccard P. The distribution of the flora in the alpine zone. 1. New phytologist. 1912;11(2):37–50.

3. M K V, K K. A Survey on Similarity Measures in Text Mining. Machine Learning and Applications: An International Journal. 2016;3:19–28. doi:10.5121/mlaij.2016.3103.

4. Hodges JL. The significance probability of the smirnov two-sample test. Arkiv för Matematik. 1958;3(5):469 – 486. doi:10.1007/BF02589501.
