# Supplementary material for: Exploring weighted network backbone extraction: A comparative analysis of structural techniques
Source: PLoS One. 2025 May 20;20(5):e0322298. doi: 10.1371/journal.pone.0322298 (PMC12091788; doi:10.1371/journal.pone.0322298)
Supplement: S4 File — (PDF) [file pone.0322298.s004.pdf]

1 Network Categories Influence on the Backbone Properties

This subsection investigates the influence of network categories on the backbone properties. To do so, for each property under investigation, we plot a heatmap with the backbone methods on the x-axis and the network categories on the y-axis. The following figures report the results of each property.

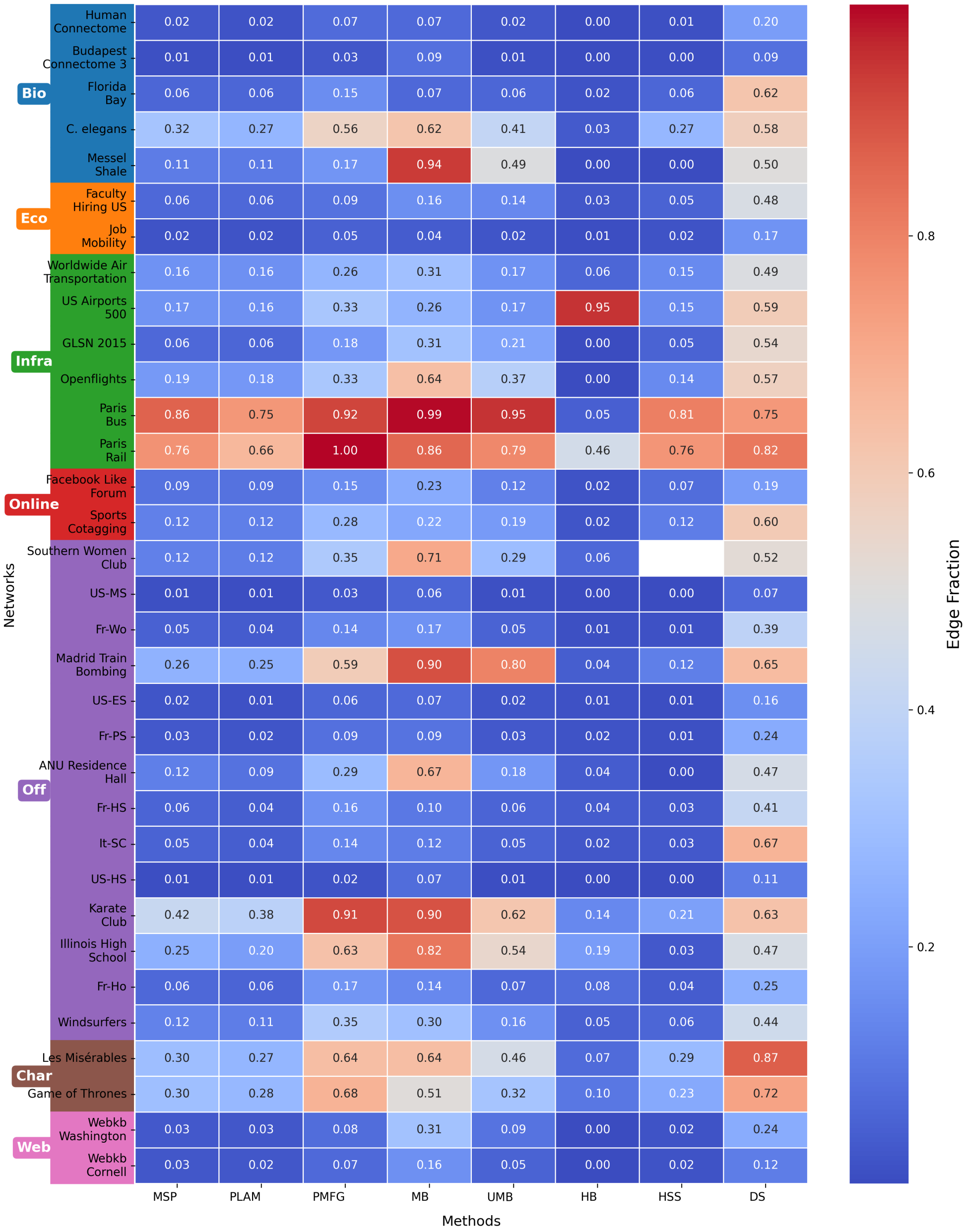

Fig 1. Heatmap of edge fraction values for backbone extraction methods across different network types. The x-axis displays the methods: Maximum Spanning Tree Filter (MSP), Planar Maximally Filtered Graph (PMFG), Primary Linkage Analysis (PLAM), h-Backbone Filter (HB), Metric Backbone (MB) and Ultrametric Backbone (UMB), Doubly Stochastic Filter (DS), and High Salience Skeleton Filter (HSS). The y-axis categorizes networks into biological, economical, infrastructural, offline social, online social, character, and web networks. The color scale represents the edge fraction of the extracted backbones.

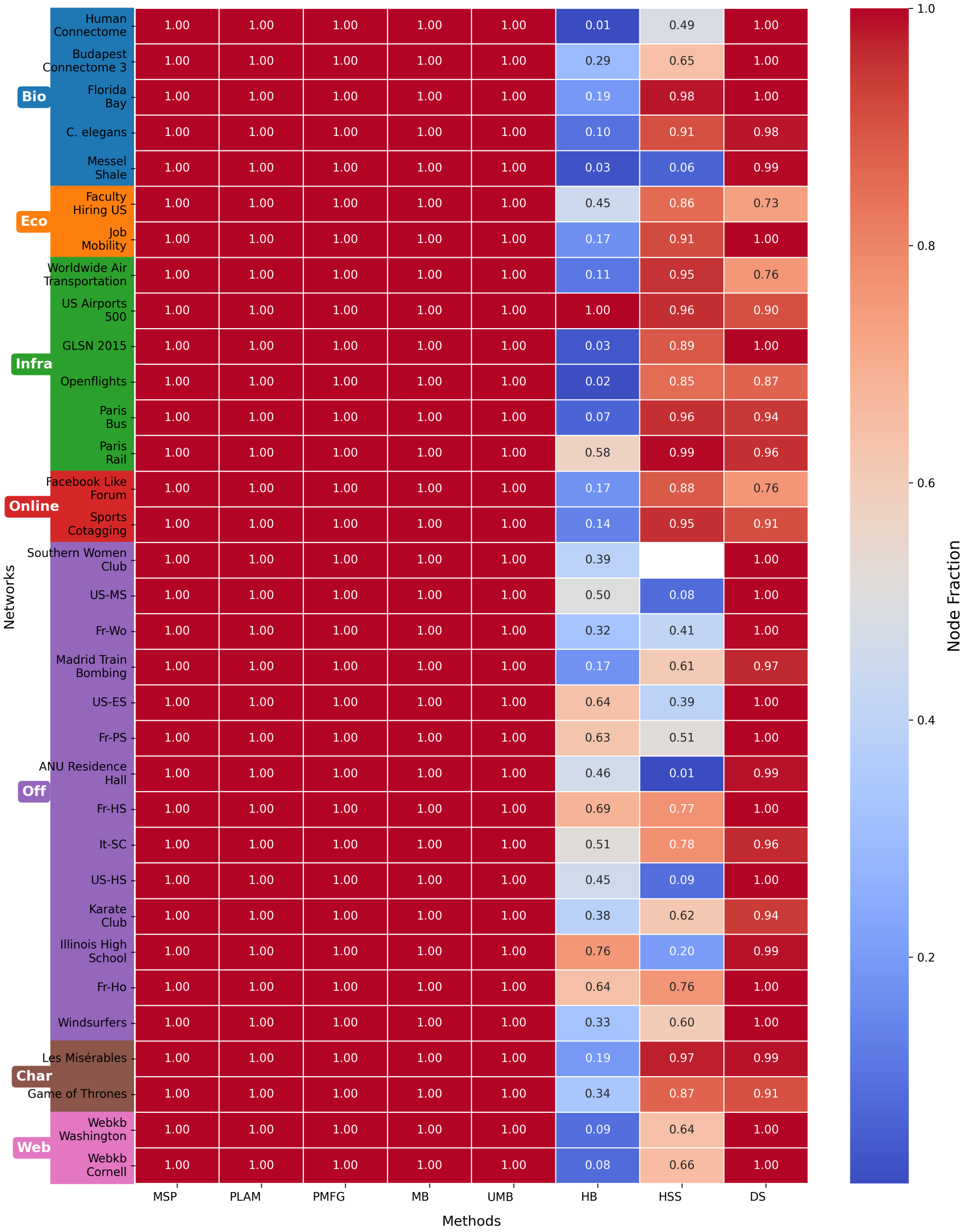

Fig 2. Heatmap of node fraction values for backbone extraction methods across different network types. The x-axis displays the methods: Maximum Spanning Tree Filter (MSP), Planar Maximally Filtered Graph (PMFG), Primary Linkage Analysis (PLAM), h-Backbone Filter (HB), Metric Backbone (MB) and Ultrametric Backbone (UMB), Doubly Stochastic Filter (DS), and High Salience Skeleton Filter (HSS). The y-axis categorizes networks into biological, economical, infrastructural, offline social, online social, character, and web networks. The color scale represents the node fraction of the extracted backbones.

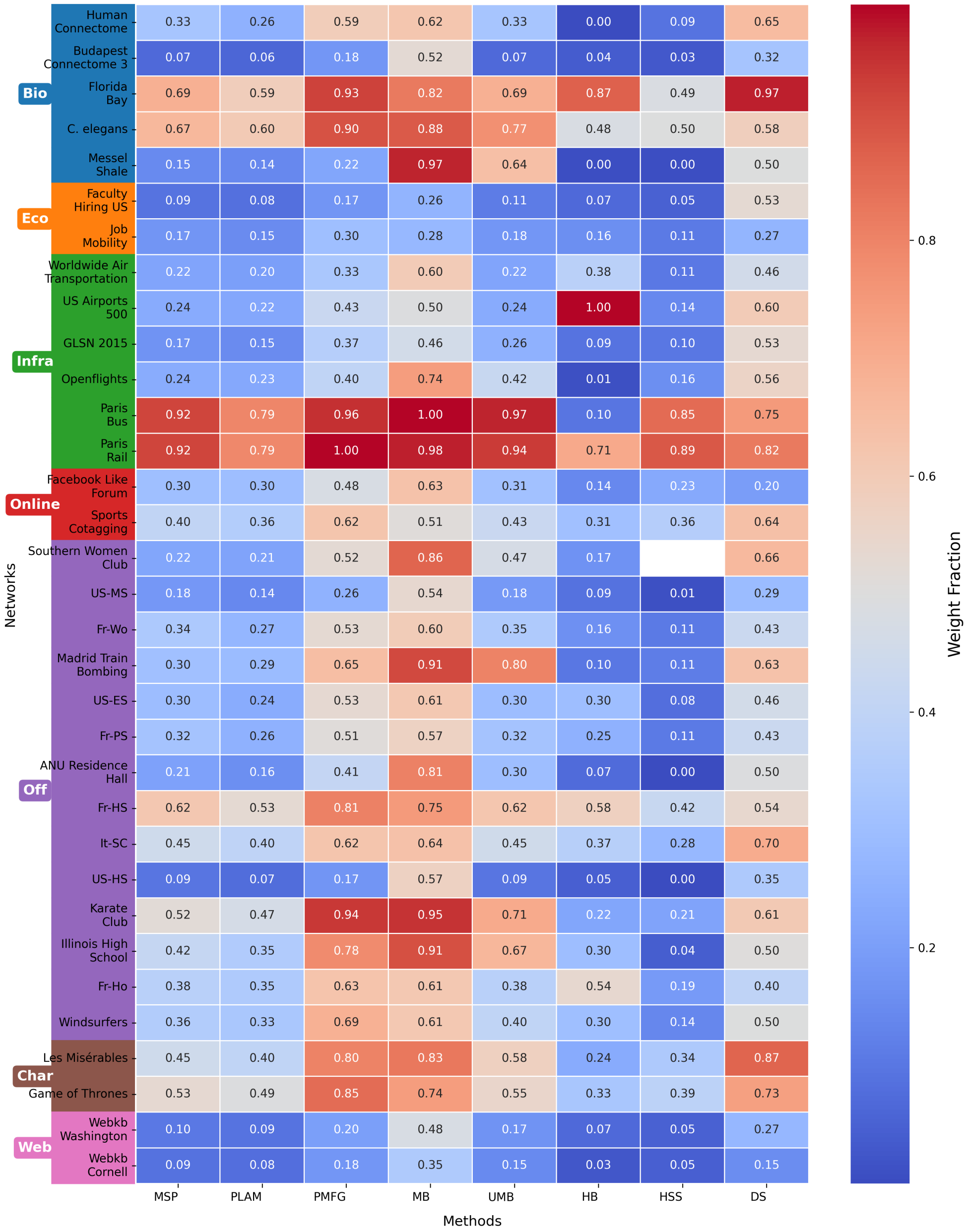

Fig 3. Heatmap of weight fraction values for backbone extraction methods across different network types. The x-axis displays the methods: Maximum Spanning Tree Filter (MSP), Planar Maximally Filtered Graph (PMFG), Primary Linkage Analysis (PLAM), h-Backbone Filter (HB), Metric Backbone (MB) and Ultrametric Backbone (UMB), Doubly Stochastic Filter (DS), and High Salience Skeleton Filter (HSS). The y-axis categorizes networks into biological, economical, infrastructural, offline social, online social, character, and web networks. The color scale represents the weight fraction of the extracted backbones.

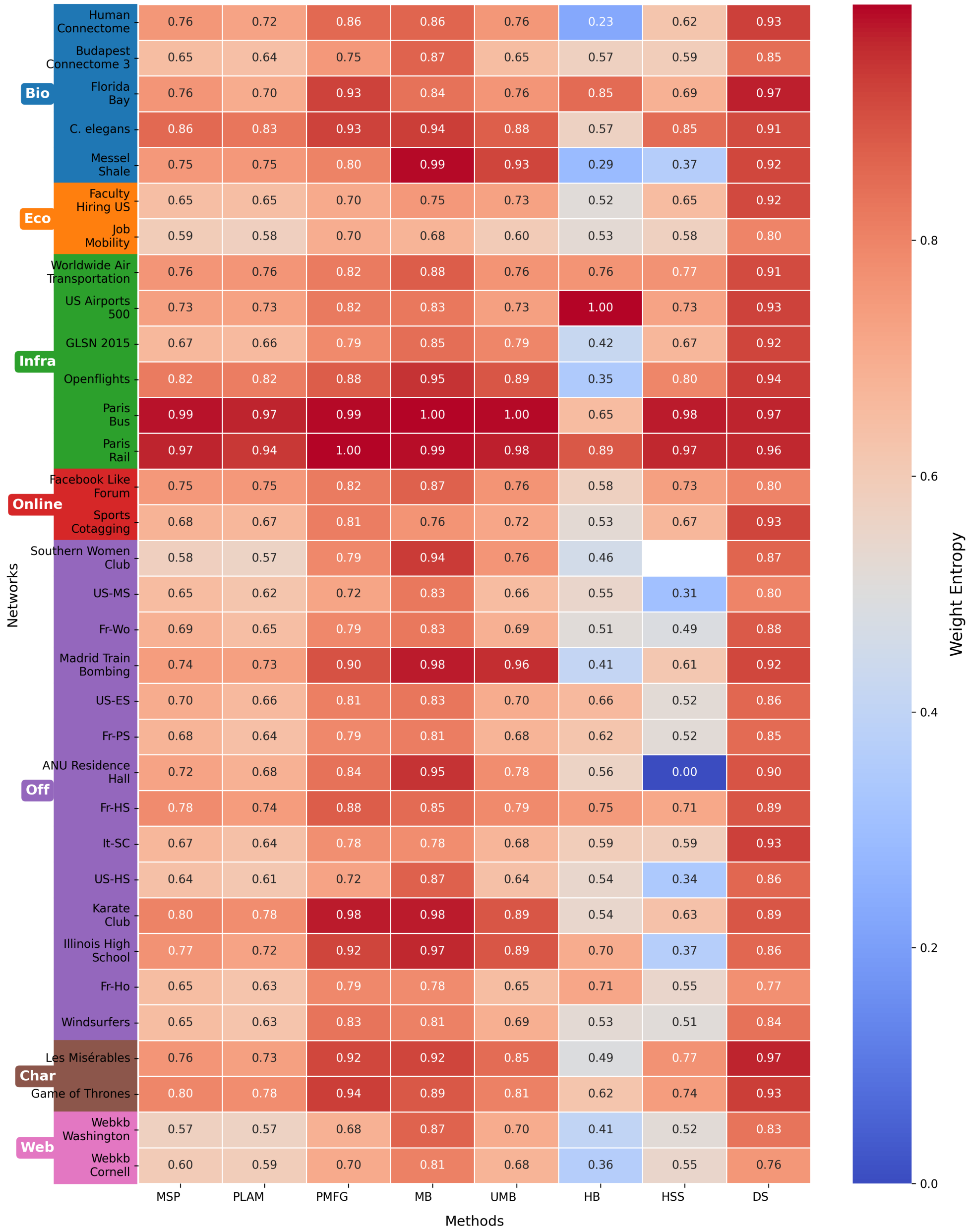

Fig 4. Heatmap of weight entropy values for backbone extraction methods across different network types. The x-axis displays the methods: Maximum Spanning Tree Filter (MSP), Planar Maximally Filtered Graph (PMFG), Primary Linkage Analysis (PLAM), h-Backbone Filter (HB), Metric Backbone (MB) and Ultrametric Backbone (UMB), Doubly Stochastic Filter (DS), and High Salience Skeleton Filter (HSS). The y-axis categorizes networks into biological, economical, infrastructural, offline social, online social, character, and web networks. The color scale represents the weight entropy of the extracted backbones.

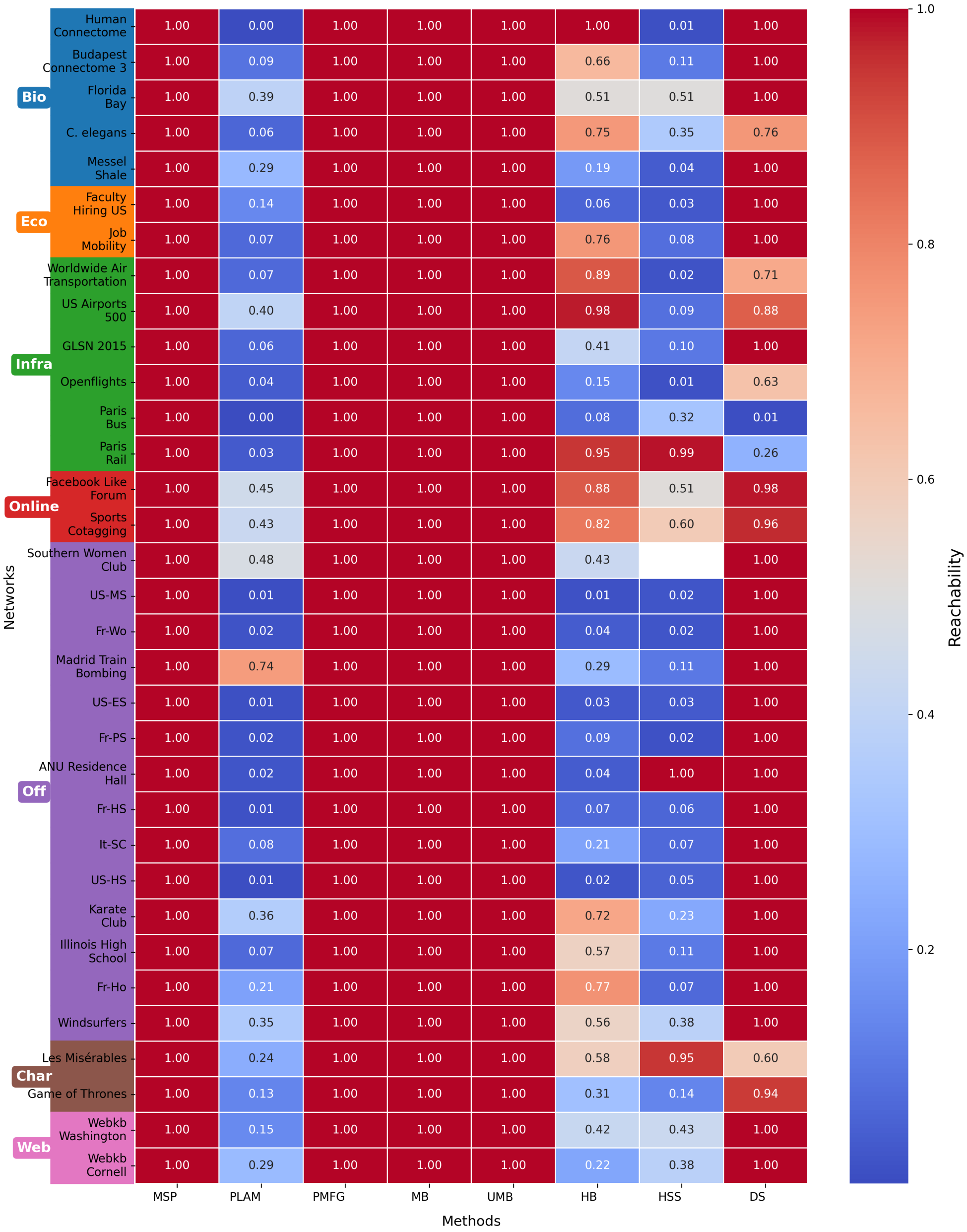

Fig 5. Heatmap of reachability values for backbone extraction methods across different network types. The x-axis displays the methods: Maximum Spanning Tree Filter (MSP), Planar Maximally Filtered Graph (PMFG), Primary Linkage Analysis (PLAM), h-Backbone Filter (HB), Metric Backbone (MB) and Ultrametric Backbone (UMB), Doubly Stochastic Filter (DS), and High Salience Skeleton Filter (HSS). The y-axis categorizes networks into biological, economical, infrastructural, offline social, online social, character, and web networks. The color scale represents the reachability of the extracted backbones.

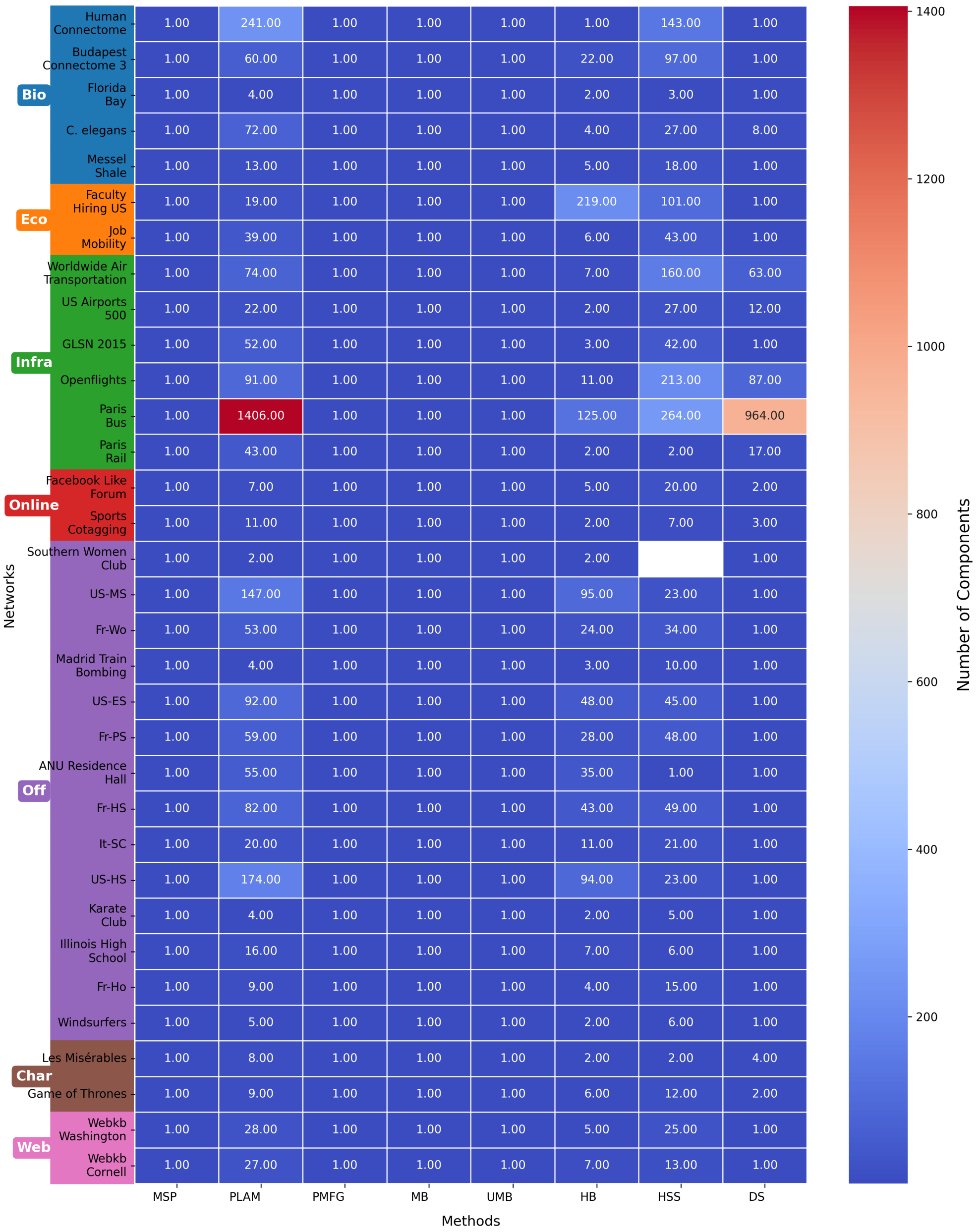

Fig 6. Heatmap of number of components values for backbone extraction methods across different network types. The x-axis displays the methods: Maximum Spanning Tree Filter (MSP), Planar Maximally Filtered Graph (PMFG), Primary Linkage Analysis (PLAM), h-Backbone Filter (HB), Metric Backbone (MB) and Ultrametric Backbone (UMB), Doubly Stochastic Filter (DS), and High Salience Skeleton Filter (HSS). The y-axis categorizes networks into biological, economical, infrastructural, offline social, online social, character, and web networks. The color scale represents the number of components of the extracted backbones.

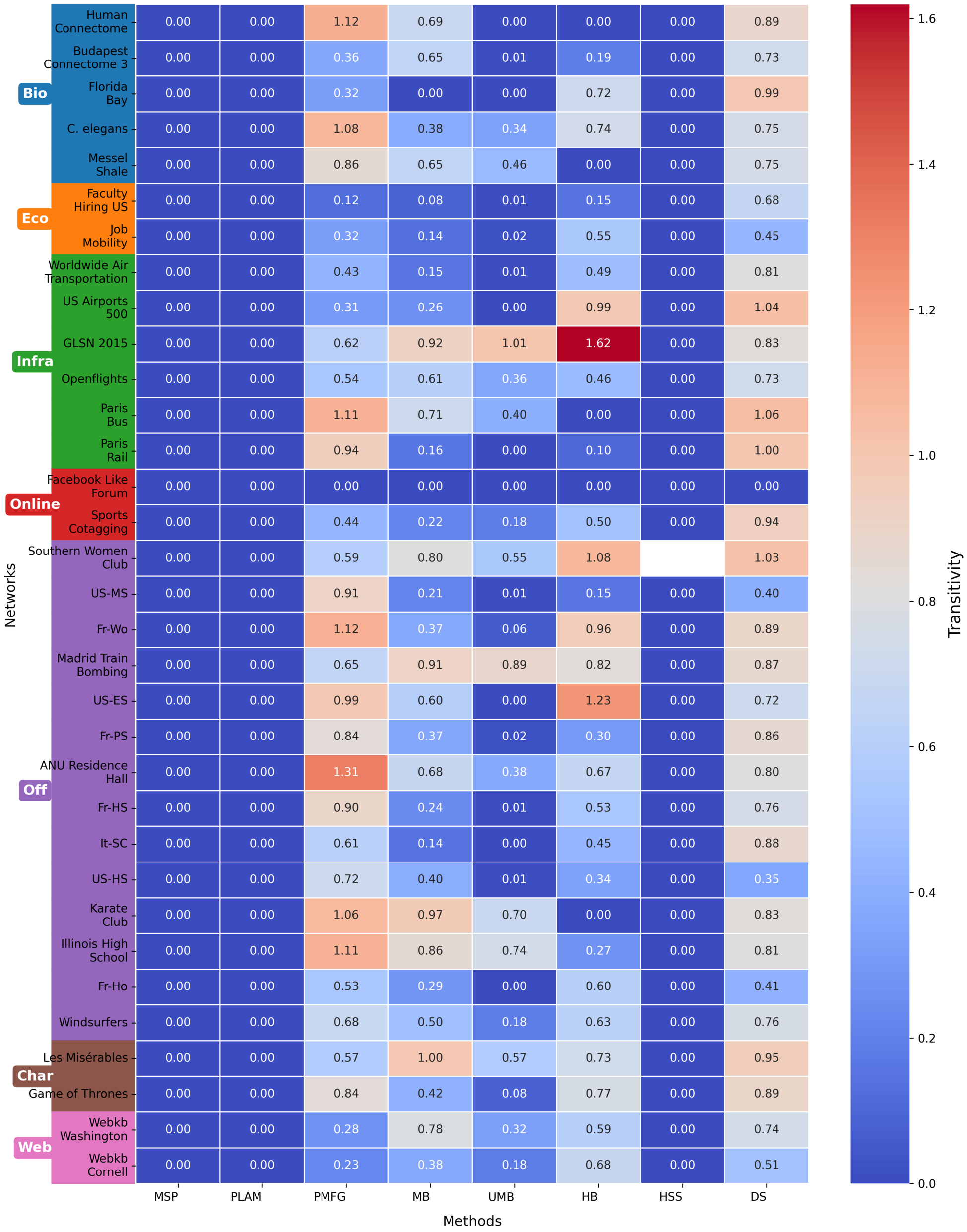

Fig 7. Heatmap of transitivity values for backbone extraction methods across different network types. The x-axis displays the methods: Maximum Spanning Tree Filter (MSP), Planar Maximally Filtered Graph (PMFG), Primary Linkage Analysis (PLAM), h-Backbone Filter (HB), Metric Backbone (MB) and Ultrametric Backbone (UMB), Doubly Stochastic Filter (DS), and High Salience Skeleton Filter (HSS). The y-axis categorizes networks into biological, economical, infrastructural, offline social, online social, character, and web networks. The color scale represents the transitivity of the extracted backbones.
